# Stereotactic cardiac radiotherapy for refractory ventricular tachycardia in structural heart disease patients: a systematic review

**DOI:** 10.1093/europace/euae305

**Published:** 2024-12-24

**Authors:** Amulya Gupta, Zeeshan Sattar, Nourhan Chaaban, Sagar Ranka, Cameron Carlson, Farhad Sami, Clifford G Robinson, Phillip S Cuculich, Seth H Sheldon, Madhu Reddy, David Akhavan, Amit Noheria

**Affiliations:** Department of Cardiovascular Medicine, The University of Kansas Medical Center, 3901 Rainbow Blvd., Mail Stop 4023, Kansas City, KS 66160, USA; Department of General and Hospital Medicine, The University of Kansas Medical Center, Kansas City, KS, USA; Department of Internal Medicine, The University of Kansas School of Medicine, Wichita, KS, USA; Division of Cardiology, Icahn School of Medicine at Mount Sinai, New York, NY, USA; University of Denver, Natural Sciences and Mathematics, Denver, CO, USA; Division of Cardiology, University of Iowa, Iowa City, IA, USA; Department of Radiation Oncology, Washington University in St. Louis, St. Louis, MO, USA; Cardiovascular Division, Washington University in St. Louis, St. Louis, MO, USA; Department of Cardiovascular Medicine, The University of Kansas Medical Center, 3901 Rainbow Blvd., Mail Stop 4023, Kansas City, KS 66160, USA; Department of Cardiovascular Medicine, The University of Kansas Medical Center, 3901 Rainbow Blvd., Mail Stop 4023, Kansas City, KS 66160, USA; Department of Radiation Oncology, The University of Kansas Medical Center, Kansas City, KS, USA; Department of Cardiovascular Medicine, The University of Kansas Medical Center, 3901 Rainbow Blvd., Mail Stop 4023, Kansas City, KS 66160, USA

**Keywords:** Cardiac, Ventricular tachycardia, VT, Stereotactic body radiotherapy, SBRT, Stereotactic ablative body radiotherapy, SABR, Stereotactic arrhythmia radioablation, STAR, Radiotherapy, Radioablation, Meta-analysis

## Abstract

**Aims:**

Among patients with structural heart disease with ventricular tachycardia (VT) refractory to medical therapy and catheter ablation, cardiac stereotactic body radiotherapy (SBRT) is a paradigm-changing treatment option. This study aims to assess the efficacy of cardiac SBRT in refractory VT by comparing the rates of VT episodes, anti-tachycardia pacing (ATP) therapies, and implantable cardioverter-defibrillator (ICD) shocks post-SBRT with pre-SBRT.

**Methods and results:**

We performed a comprehensive literature search and included all clinical studies reporting outcomes on cardiac SBRT for VT. Treatment efficacy was evaluated as random-effects pooled rate-ratios of VT episodes, ATP therapies and ICD shocks post-SBRT (after 6-week blanking) and pre-SBRT, with patients serving as their own controls. Post-SBRT overall survival was assessed using Kaplan–Meier method. We included 23 studies published 2017–24 reporting on 225 patients who received cardiac SBRT, with median follow-up 5.8–28 months. There was significant heterogeneity among the studies for all three efficacy endpoints (*P* < 0.00001). The random-effects pooled rate-ratios of VT episodes, ATP therapies and ICD shocks post- vs. pre-SBRT were 0.10 (95% CI 0.06, 0.16), 0.09 (0.05, 0.15), and 0.09 (0.05, 0.17), respectively (all *P* < 0.00001). The most common reported complications included pericardial (8.0%, including 0.9% late oesophagogastro-pericardial fistula) and pulmonary (5.8%). There was no change in left ventricular ejection fraction post-SBRT (*P* = 0.3) but some studies reported an increase in mitral regurgitation. The combined 3-, 12-, and 24-month overall patient survival was 0.86 (0.80, 0.90), 0.72 (0.65, 0.78), and 0.57 (0.47, 0.67), respectively.

**Conclusion:**

Among patients with refractory VT in context of structural heart disease, VT burden and ICD shocks are dramatically reduced following cardiac SBRT. The overall mortality in this population with heart failure and refractory VT receiving palliative cardiac SBRT remains high.

## Introduction

Ventricular tachycardia (VT) in patients with structural heart disease is a life-threatening heart rhythm disorder. It is primarily caused by electrical re-entry within and around regions of heterogeneous myocardial fibrosis. An implantable cardioverter-defibrillator (ICD) can prevent VT-related sudden death by rapid identification and automated treatment of VT with anti-tachycardia pacing (ATP) or ICD shock.^1^ However, ICD shocks diminish the quality of life and have an adverse effect on long-term outcomes.^2,3^

Antiarrhythmic drug therapy is the first line of treatment to suppress VT but has modest efficacy and is associated with adverse effects.^4^ Ventricular tachycardia circuits harboured within regions of myocardial scar can be treated by catheter ablation.^5,6^ However, VT catheter ablation is associated with high morbidity and mortality and yet VT recurrences can occur.^7–9^ Patients with recurrent VT despite antiarrhythmic drugs and catheter ablation have limited therapeutic options and are at a high risk for mortality.^5^

Stereotactic body radiotherapy (SBRT) or stereotactic ablative radiotherapy (SABR) delivers high doses of electromagnetic radiation precisely to targets in the body and is widely available for cancer treatment. Cardiac SBRT, radiosurgery, or stereotactic arrhythmia radioablation (STAR) is a paradigm-changing treatment option for refractory VT which delivers therapeutic photons non-invasively to the arrhythmogenic substrate.

One of the first-in-human case reports of cardiac SBRT with 25 Gy delivered in a single fraction for treatment of VT was published in 2015.^10^ The first human case series on compassionate use of cardiac SBRT was published in 2017 by Cuculich *et al*.,^11^ showing a marked reduction in VT burden in five patients with refractory VT. The same group, Robinson *et al*.,^12^ then published the first prospective uncontrolled trial of cardiac SBRT in 19 patients. Since then, several uncontrolled studies have been reported describing mostly single-centre experiences.^13–33^ Cardiac SBRT still remains a novel treatment modality lacking evaluation in a randomized controlled trial. Therefore, we sought to review the pooled efficacy of this therapy as reported in all the published studies.

## Methods

### Data sources and searches

We performed a systematic literature search on PubMed up to March 2024 to identify relevant studies (see Supplementary material for details). A similar search was executed in Google Scholar to ensure completeness. We looked through the references of the included studies and review papers to identify any missing studies. We also searched for any subsequent abstract presentations or publications from these studies reporting on longer-term outcomes. We searched clinicaltrials.gov for condition/disease ‘ventricular tachycardia’ and intervention/treatment ‘radiotherapy’. Given the public availability of data, this study is exempt from Institutional Review Board approval.

### Study selection

We conducted this study per the Preferred Reporting Items for Systematic Reviews and Meta-Analyses (PRISMA) statement. Following inclusion criteria were used: (i) studies including humans (≥3) with VT, (ii) undergoing cardiac SBRT treatment, and (iii) reporting VT recurrences, ICD therapies, and/or long-term events. Duplicate studies, meta-analyses, or review articles were excluded. Case reports were not included but were separately compiled in the Supplementary material.

### Data extraction

We extracted data from all included studies on a standardized worksheet. The following baseline variables were collected: study authors, title, year and journal of publication, methodology, demographics, clinical information, and prior VT therapies. The following SBRT characteristics were collected: planning treatment volume, number of left ventricular segments treated, radiation dose, and adverse events/complications. Outcome efficacy data were collected as the number of patients, total patient-months of follow-up, and the total number of reported (i) VT episodes, (ii) ATP therapies, and (iii) ICD shocks combined for all study patients post-SBRT (after 6-week blanking) vs. pre-SBRT. If the study had a different blanking period, then we checked if the data provided allowed us to recalculate results using 6-week blanking period, failing which we had to resort to next closest option for the blanking period (0–3 months). We also collected information on post-SBRT antiarrhythmic drug therapy use and serious adverse events. For combined time-to-event outcomes of (i) overall survival, (ii) recurrent VT-free survival, (iii) ICD shock-free survival, and (iv) repeat catheter ablation/SBRT-free survival, data were manually extracted from the text, figures, and/or supplement of the publications (see Supplementary material for detailed methodology).

### Missing data

In case of absence of specific data points, contextual imputation of data was done. When number of total VT episodes or ATP therapies was not available, the respective missing values were imputed assuming total VT episodes = ATP therapies + ICD shocks. We also performed separate analysis for rate-ratios of VT episodes and ATP therapies using as reported events without imputing missing data. For calculating weighted averages across studies of variables like left ventricular ejection fraction, when only median (range or interquartile range [IQR]) was provided, mean ± SD was imputed from the provided *n*, median, and range/IQR using an online calculator which uses statistically validated equations for this conversion.^34^

### Endpoints

The efficacy of cardiac SBRT was evaluated as the rate ratio of (i) VT episodes, (ii) ATP therapies, and (iii) ICD shocks post-SBRT (after blanking) as compared with pre-SBRT with the patients undergoing SBRT being their own controls. Kaplan–Meier (KM) curves were generated to depict the (i) overall survival, (ii) VT recurrence-free survival (after blanking period), (iii) ICD shock-free survival (after blanking period), and (iv) repeat catheter ablation/SBRT-free survival post-index SBRT.

### Statistical analyses

Weighted averages were used to summarize the baseline variables from different studies. The efficacy endpoints were pooled as the rate-ratios using the random-effects method and displayed as forest plots. For studies with a zero event rate for any of three VT outcomes in either arm, we used 0.5 for continuity correction. We evaluated inter-study heterogeneity using the *I*^2^ statistic. Sensitivity analysis was done to evaluate how removal of each study affected the overall outcome. To address publication bias, we used visual inspection of funnel plots. Separate sensitivity analyses were performed for the efficacy outcomes after removing the outlier studies identified from the funnel plots. Two-sided *P*-value 0.05 was considered the threshold for statistical significance. Review Manager (RevMan), Version 5.4, The Cochrane Collaboration, 2020 was used to obtain the forest and funnel plots. For each KM time-to-event analysis following SBRT, data were collected by combining all patients from all studies in one group that underwent cardiac SBRT. Kaplan–Meier survival analyses were performed in R version 4.4.1.

## Results

### Study population

The primary included studies, detailed inclusion criteria, demographics, and relevant clinical data are summarized in *Table 1*. We included 23 studies (see Supplementary material online, *Figure S1*), published 2017–24, reporting on 202 patients who underwent cardiac SBRT. Additionally, Wight *et al*.^35^ reported long-term outcomes on Lloyd *et al*. (*n* = 10) adding another four patients (*n* = 14). Hašková *et al*. reported efficacy outcomes on 17 patients but added another 19 (*n* = 36) for safety outcomes. So, the total number of patients represented in this systematic review is 202 + 4 + 19 = 225. Of these, 191 patients from 21 studies were available for efficacy meta-analysis of VT events (Molon *et al*. and Chang *et al*. did not provide data), and 200 patients from 22 studies for overall survival analysis. In addition, we have complied a separate list of case reports reported in the literature comprising 32 individual cases (see Supplementary material online, *Table S1A* and *B*), bringing the total experience captured in this manuscript to 257 cases.

**Table 1:** Summary of baseline characteristics of studies reporting ventricular tachycardia outcomes with cardiac SBRT^a^

Number	First author, year	Type of uncontrolled study	*N*	Inclusion criteria	Age (in years)	Sex	Heart disease	NYHA class	Antiarrhythmic drug therapy	Number of catheter ablations	Number of VTs induced/targeted
1	Cuculich P. Dec. 2017^11^	Retrospective case series	5	≥3 episodes of ICD-treated VT ≥2 AAD ≥1 CA or contraindicated for CA	66 ± 10 (60–83)	4 M 1 F	2 ICM 3 NICM	3.8 (3–4)	5 Amiodarone 5 Mexiletine	1.4 (1–4)	2.8 (14 total)
2	Robinson C. Jan. 2019^12^	Prospective uncontrolled trial	17^b^	Age ≥18 years Refractory sustained monomorphic VT (≥3 episodes) ≥1 AAD ≥1 CA or contraindicated for CA	Median 66 (49–81)	17 M 2 F	11 ICM 8 NICM	2.9 (1–4)	10 High dose Amiodarone 2 Low dose Amiodarone 11 Class I 7 Class III	1.5 (29 total; Endo-25/Epi-4)	2
3	Neuwirth R. Jul. 2019^13^	Retrospective case series	10	Scar-related VT Inducible during programmed electrical stimulation Failed CA	66 ± 7 (61–78)	9 M 1 F	8 ICM 2 NICM	2.4 (2–3)	10 Amiodarone	Endo 1.8 (1–4) Epi 0.4 (0–1)	N/A
4	Lloyd M. Mar. 2020^14^ (Wight J. Apr. 2022)^35^	Retrospective case series	10 14	≥2 AAD ≥1 CA Failed 1 adjunctive therapy (mechanical support/sympathetic blockade)	62 ± 9 (50–78)	7 M 3 F	4 ICM 6 NICM (incl. 1 post-viral myocarditis, 1 sarcoidosis) 3 LVAD	N/A	2.0 (1–3) 8 Amiodarone 5 Mexiletine 2 Sotalol 3 Lidocaine 1 Quinidine 1 Phenytoin	2.0 (1–5)	2.2 ± 2.2 (1–>7) morphologies
5	Gianni C. Aug. 2020^15^	Prospective uncontrolled trial	5	Age ≥60 years Recurrent VT and ICD shocks Failed CA and AAD LVEF ≥20%	63 ± 12 (45–76)	5 M	4 ICM 1 NICM	1.8 (1–2)	5 Amiodarone	1.6 (1–2)	N/A
6	Ho L. May. 2021^16^	Retrospective case series	6^c^	Age ≥18 years ≥3 Sustained VTs in 3 months Failed AAD ≥1 CA or contraindicated for CA	55 ± 18 (23–80)	6 M 1 F	1 ICM 3 DCM 1 HCM 1 ARVCw 1 PVC	N/A	6 Amiodarone	1.7 (0–4)	N/A
7	Yugo D. Jun. 2021^17^	Retrospective case series	3	Recurrent VT and ICD shocks Failed CA and AAD	72 ± 10 (65–83)	2 M 1 F	3 NICM (antero-septal)	1.3 (1–2)	2 Amiodarone 2 Mexiletine 1 Lidocaine	1.3 (1–2)	3.7 (2–5)
8	Chin R. Sep. 2021^18^	Retrospective case series	8	Refractory VT ≥1 CA or contraindicated for CA Contraindicated for advanced HF therapies	75 ± 7 (65–86)	8 M	4 ICM 4 NICM	3.4 (3–4)	6 Amiodarone 4 Mexiletine 1 Sotalol 1 Lidocaine 3 Ranolazine	1.6 (0–5) 4 Epi	1.1 (1–2)
9	Ho G. Sep. 2021^19^	Retrospective case series	6	Refractory VT failed AAD, CA, stellate ganglion block	74 ± 6 (64–81)	6 M	2 ICM 4 NICM	3.7 (3–4)	2.2 ± 1.1 (failed 1–5 AAD)	2.2 (1–3)	4.0 (1–7)
10	Carbucicchio C. Nov. 2021^20^	Prospective uncontrolled trial	7^d^	Age ≥50 years Refractory VT ≥3 ICD therapies LVEF ≥20% NYHA Class II–III	70 ± 7 (59–78)	7 M	3 ICM 4 NICM	2.7 (2–3)	2.7 (2–3)	Endo 1.6 (0–3) Epi 0.4 (0–2)	N/A
11	Lee J. Nov. 2021^21^	Retrospective case series	7	Recurrent VT Failed AAD ≥1 CA or contraindicated for CA	73 ± 4 (68–78)	4 M 3 F	5 ICM 2 NICM (incl. 1 myocarditis)	2.7 (2–4)	1.1 (1–2) 7 Amiodarone 2 Mexiletine 1 Propafenone 2 Ranolazine	1.9 (0–3)	1.7 (1–3)
12	Qian P. Jan. 2022^22^	Retrospective case series	6	Ischaemic CMP VT refractory to AADs and CA	Median 72 (IQR 70–73)	6 M	6 ICM	Median 2 (IQR 2–2.75)	Median 2 (IQR 2–2.75) 6 Amiodarone 6 Quinidine 5 Mexiletine 1 Sotalol 1 Ranolazine	Median 2 (IQR 2–3.5) 2 Epi	Median 2 (IQR 0.25–3.75)
13	Molon G. May 2022^23^	Prospective case series	6	Age >18 years ICD ≥6 months ≥3 VT episodes with ICD therapy Not eligible for CA or failed CA Failed AAD	75 ± 10 (61–85)	5M 1F	4 ICM 2 NICM	2.8 (2–4)	5 Amiodarone 1 Mexiletine	2 (0–1)	N/A
14	Aras D. Aug 2022^24^	Prospective case series	8	Age ≥18 years and ≥3 VT episodes in 6 months ≥1 CA, ≥3 VT episodes in 24 h ≥1 CA, or CA contraindicated	58 ± 14 (46.5–78.5)	8 M	2 ICM 4 NICM 1 DCM 1 HCM	2.8 (2–3)	7 Amiodarone 4 Mexilitine 1 Sotalol	N/A	N/A
15	Ninni S. Sep 2022^25^	Retrospective case series	17	Electrical storm (≥3 VT episodes in 24 h)	67 ± 13	13M 4F	10 ICM 4 DCM 1 sarcoidosis 1 LVNC 1 congenital	1.9 (1–3)	17 Amiodarone 10 Lidocaine	1.5 (0–4)	N/A
16	Chang W. Dec 2022^26^	Prospective case series	5^c^	Age ≥19 years >2 documented VTs Or ICD shock or ATP due to VTs	72 ± 7.4	4 M 1 F	2 NICM 3 ICM	3.4 (3–4)	3 Carvedilol 3 Bisoprolol 5 Amiodarone	0.8 (0–2)	N/A
17	Ree M. Feb 2023^27^	Prospective uncontrolled trial	6	Age >18 years ICD Refractory VT	Median 73 (54–83)	6M	6 ICM	2.5 (2–3)	5 Amiodarone 5 Mexilitine	Median 2 (1–5) 10 Endo 2 Epi	ICD VT morpholgies 9 (3–14)
18	Amino M. Feb 2023^28^	Interim report of prospective uncontrolled trial	3	>3 VT episodes	74 ± 15 (60–91)	1M 2F	2 ICM 1 HCM	3 ± 1 (2–4)	3 Amiodarone	0.3 (0–1)	4.5 ± 3.5 (2–7)
19	Krug D. Jul. 2023^29^	Interim report of prospective uncontrolled trial	5	Age ≥18 years SHD ICD VT	64 ± 9 (49–74)	4 M 1 F	2 ICM 2 NICM 1 HCM	N/A	5 Amiodarone 3 Mexiletine 1 Lidocaine	3 (0–6)	N/A
20	Herrera Siklody C. Oct. 2023^30^	Retrospective case series	20	Refractory VT 16/20 Electrical storm	Median 68 (47–80)	15 M 5 F	6 ICM 9 NICM 4 Inflammatory 1 Cardiac metastasis	N/A	17 Amiodarone 3 Sotalol 2 Lidocaine 3 Mexiletine 4 Flecainide 1 Propafenone	2 (0–6) 5 Epi	Median 5.5 (4–11)
21	Miszczyk M. Nov. 2023^31^	Prospective trial	11	Age ≥ 18 years SHD ICD VT with pharmacological management, at least 1 previous CA or contraindication to CA	Median 67 (45–72)	10 M 1 F	9 ICM 2 NICM: 1 Peripartum cardiomyopathy 1 inflammatory cardiomyopathy	2.1(1–3)	4 Amiodarone 4 Mexiletine	2 (1–4)	N/A
22	Arkles J. Jan. 2024^32^	Prospective 1 centre registry	14	Patients with VT refractory/not suitable for AAD/ablation therapy including acutely unsuccessful CA and inducible VT in NIPS	65. ± 7.8	13 M 1F	7 ICM 7 NICM	N/A	14 Amiodarone	Mean 2.1	4.7 ± 2.1
23	Hašková J. Feb. 2024^33^	Retrospective 2 centre case series	17^e^	Recurrent scar-related VT ≥ 2 CA	65 ± 11	15 M 2 F	5 ICM 12 NICM	2.2 ± 0.5	13 Amiodarone 3 Sotalol	2.2 (1–4) 10 Epi	N/A
**Median** **(range) of all studies^f^**	**23 uncontrolled studies**	**Retrospective series (13), Prospective series (4) and Prospective trials (6)**	**7**		**67 (45–91)**			**2.7** (**1–4)**	**1.5 (0.7–2.7)**	**2**(**0–6)**	**3.3 (1–14)**
**Patient aggregates/weighted averages**			**202**	**All refractory VT**	**Avg. 67**	**175 M (85%)** **30 F (15%)**	**106 ICM (52%)** **99 NICM (48%)**	**Avg. 2.6^g^**	**Avg. 1.5**	**Avg. 1.9^g^**	**Avg. 3.6^g^**

AAD, antiarrhythmic drug; avg., average; CA, catheter ablation; DCM, dilated cardiomyopathy; Endo, endocardial; Epi, epicardial; F, female; HCM, hypertrophic cardiomyopathy; ICD, implantable cardioverter-defibrillator; ICM, ischaemic cardiomyopathy; LVNC, left ventricular non-compaction; M, male; NICM, non-ischaemic cardiomyopathy; N/A, not available; SHD, structural heart disease; VT, ventricular tachycardia; IQR, interquartile range.

^a^Absolute number or average ± SD/range is provided unless specified.

^b^Excluding two patients treated for PVCs.

^c^Excluding one patient treated for PVCs.

^d^Excluding one patient who did not receive SBRT.

^e^Efficacy cohort 17 patients, safety cohort 36 patients.

^f^Bolded row provides summary of the findings from the included studies.

^g^Excluding not available.

Of the included studies, 10 were prospectively planned. The average age of the included patients was 67 years, with individual patients ranging 45–91 years. Majority of the treated patients (85%) were male. Ischaemic cardiomyopathy was present in 52% patients. The average NYHA functional class was 2.6. All patients had refractory VT and had failed antiarrhythmic drugs. The average number of previous catheter ablations was 1.9 with individual patient range 0–6. Many studies mentioned cardiac SBRT was offered as a last-resort or palliative option. Number of VT morphologies induced/targeted ranged 1–14 (average 3.6).

### Cardiac stereotactic body radiotherapy

Details of cardiac SBRT treatment, follow-up and adverse outcomes are shown in *Table 2*. Patients received 25 Gy dose in a single fraction. Slightly lower doses 22.2 Gy and 23 ± 2 Gy were used in two studies.^18,30^ The average planning treatment volume was 84 mL. The volume in individual patients ranged widely from 13 to 444 mL, while the average volume across studies ranged 23–308 mL. When reported, the number of targeted American Heart Association-defined left ventricular segments (out of 17) averaged 3.5 with individual patients ranging 1–8. The median study follow-ups ranged 5.8–28 months, with average follow-up 13.3 months.

**Table 2:** Details of cardiac SBRT treatment and adverse outcomes reported in the included studies^a^

Number	First author, year	Planning treatment volume (mL)	Left ventricular segments	Radiation dose^b^	Treatment time (min)	Follow-up (months)	AAD/CA during follow-up	Significant adverse events	Deaths
1	Cuculich P. 2017 (*n* = 5)	49 (17–81)	N/A	25 Gy	On-table 14 (11–18)	12	1 Resumed Amiodarone 1 Repeat CA	None	1 Died (in 12 months)
2	Robinson C. 2019 (*n* = 19)	Median 99 (61–299)	3.9 ± 2.0 Median 4 (1–6)	25 Gy	Beam-on Median 15.3 (5.4–32.3)	Median 13	AADs stopped in 3 pts, Decrease in no. and dose of AADs overall	2 Pneumonitis 5 Pericardial effusion 1 HF exacerbation 1 Pericarditis 1 Gastropericardial fistula 1 Late pericardial effusion	5 Died (in 12 months) 1 Unrelated
3	Neuwirth R. 2019 (*n* = 10)	23 ± 5 (14–30)	N/A	25 Gy	Total 68 (45–80)	Median 28 (16–54)	2 Resumed Amiodarone	1 Increase in mitral regurgitation	3 Died (18, 43, 54 months) 1 Unrelated
4	Lloyd M. 2020 (*n* = 10), Wight J. 2022 (*n* = 14)	81 ± 60 (29–238)	N/A	25 Gy	Total < 30 min	5.8 (3.9–9.0) (excl. 2 that went hospice)	No change	1 Slow VT during SBRT 4 Pneumonitis (out of 14 patients)	7 Hospice/death (out of 14)
5	Gianni C. 2020 (*n* = 5)	143 ± 50 (80–184)	N/A	25 Gy	Total 82 ± 11 (66–92)	12	Decrease in no. and dose of AADs 3 Repeat CA	None	2 (10, 12 months)
6	Ho L. 2021 (*n* = 7)	54 ± 31 (14–93)	N/A	25 Gy	Beam-on 12.8 ± 2.6 (9.2–17.3)	Median 14.5	N/A	1 Pericardial effusion	1 Unrelated
7	Yugo D. 2021 (*n* = 3)	83 ± 22 (64–107)	N/A	25 Gy	Total 73 ± 55 (20–130)	13.5 ± 2.8	Continued AADs	1 Pneumonitis unrelated	3 (1, 13, 14 months) 1 Unrelated
8	Chin R. 2021 (*n* = 8)^c^	103 ± 56 (21–191)	N/A	22.2 (range 15–25) Gy	Beam-on 17.5 ± 5.9 (10.7–26.7)	Median 7.8 (IQR 4.8–9.9)	2 Off AAD 1 Repeat SBRT (different location) 1 Sympathectomy	None	3 Died 2 Unrelated
9	Ho G. 2021 (*n* = 6)	119 ± 46 (66–193)	2.3 ± 0.8 Median 2.5 (1–3)	25 Gy	Beam-on 7.7 ± 3.1 (7.4–16.1)	6.0 ± 4.9	2 Dose reduction Amiodarone	1 Pericardial effusion	2 Died 1 Unrelated
10	Carbucicchio C. 2021 (*n* = 7)	183 ± 53 (88–239)	N/A	25 Gy	N/A	Median 8	2 Dose reduction Amiodarone 1 Off Mexiletine	1 Pulmonary fibrosis (asymptomatic)	3 Died 2 Unrelated
11	Lee J. 2021 (*n* = 7)^d^	95 ± 29 (58–139)	3.3 ± 1.1 Median 3 (2–5)	25 Gy (1 received 20 Gy)^e^	Beam-on 7.7 ± 3.1 (5–12)	Plan 6	3 Off Amiodarone 2 Dose reduction Amiodarone 2 AAD escalation 1 Repeat CA 7 weeks	None	3 Died (1,1,9 months)
12	Qian P. 2022 (*n* = 6)	308 ± 94 (171–444)	N/A	25 Gy	Beam-on 13.8 ± 3.8 (9.5–19.9)	Median 7.6 (IQR 7.0–10.2)	Decrease in AAD no. from median of 2–1.5 per patient 4 Repeat CA	1 HF exacerbation 1 Pneumonia 1 Pericardial effusion, asymptomatic	3 Died (4.4, 7.1, 8.7 months)
13	Molon G. 2022 (*n* = 6)	N/A	N/A	25 Gy	N/A	8.7 ± 6.6	N/A	N/A	1 (1 month)
14	Aras D. 2022 (*n* = 8)	Median 157.4 (70.5–272.7)	4.6 ± 1.5 Median 5 (2–6)	25 Gy	Median ablation time 5.6 (3.6–7.45)	Median 8 (1–14 months)	N/A	2 Pericardial effusions	4 Died
15	Ninni S. 2022 (*n* = 17)	Median 52 (40–64)	3.9 ± 1.4 4 (2–7)	5 received 25 Gy 12 received 20 Gy	Total Median 67 (45–70)	Median 12.5 (10.5–17.8)	1 Repeat CA	1 Pericardial effusion (asymptomatic) 1 Pneumonitis (asymptomatic)	4 Died 1 Unrelated
16	Chang W. 2022 (*n* = 5)	95.4 ± 90.8	N/A	25 Gy	Total 24.5 (5.6–77.4)	Median 12.3	Decrease in number of AADs in 2 patients	2 HF exacerbation	1 Died
17	Ree M. 2023 (*n* = 6)	Median 187(93–372	Median 5 (1–8)	25 Gy	Median Beam-on 4.6 (3.6–5.2)	12 months	Decrease in AAD dose in 4 patients	1 ICD reset during SBRT 1 Myocardial injury 2 Pericardial effusion 2 Pneumonitis 1 Intracardiac thrombus	2 Died Unrelated
18	Amino M. 2023 (*n* = 3)	67 ± 26 (50–96)	3.0 ± 0	25 Gy	Beam-on 3.6 ± 1.4 (2.6–5.2)	Mean 14	2 off Amiodarone 1 Dose reduction in Amiodarone	2 Pericardial effusions	None
19	Krug D. 2023 (*n* = 5)	64 ± 19 (43–81)	N/A	25 Gy	Total 29.0 ± 21.1 (9–61)	Median 6 (1–14)	1 Repeat CA	2 HF exacerbation 1 increase in Mitral regurgitation	2 (3 days, 7 weeks)
20	Herrera Siklody C. 2023 (*n* = 20)	Median 26 (14–115)	Median 2 (1–6)	23 ± 2 Gy	N/A	Median 25 months (0.1–47.6)	12 Repeat CA	1 Electrical storm 1 Pericardial fibrosis 1 Spontaneous rib fracture 1 Fast progression to severe aortic stenosis	7 Died
21	Miszczyk M. 2023 (*n* = 11)	Median 73 (18.6–111.3)	N/A	25 Gy	Beam on 13.4 (9.4–18.9)	Median 22.2 (1.3–28.6)	3 CA	1 HF	3 Died
22	Arkles J. 2024 (*n* = 14)	45.6 (84.7–124.1)	N/A	25 Gy	Beam on 3.5 (2.6–4.6)	9.3 ± 4.6	Decrease in Amiodarone dose from 400 ± 174.8 to 191 mg 225 ± 191mg 1 CA	1 Aspiration pneumonia	4 Died
23	Hašková J. 2024 (*n* = 36)^f^	Median 39 (13–91)	N/A	25 Gy	Total median 58 (42–82)	13.7 ± 11.6	Repeat CA: 2 (1 CA) 4 (3 CA) 2 (4 CA)	4 Lung fibrosis in small area 8 Progression of mitral regurgitation 1 Tricuspid regurgitation 2 Oesophagitis 1 Oesophago-pericardial fistula	18 Died^g^
**Median (range) of all studies^h^**	**23 studies**	**82 (13–444) mL**	**3.6 (1–8)**	**25 Gy**	**10.3 (2.6–32.3) min**	**12 months** **(range of medians 5.8–28)**			
**Patient aggregates/weighted averages**	***n* = 225**	**Avg.^i^ 84 mL**	**Avg.^i^ 3.5**	**Predominantly single fraction 25 Gy**	**Avg. beam-on time^i^ 11.3 min**	**Avg. 13.3 months**	**Most with reduction in AAD** **Most continued amiodarone** **Some had repeat CA**	**Avg. 0.28** **13 (5.8%) Lung-related** **18 (8.0%) Pericardium related incl.** **2 (0.9%) GI-pericardial fistulas** **10 (4.4%) progression of mitral regurgitation**	**82/225^j^ (36%)**

HF, heart failure; other abbreviations as mentioned in *Table 1*.

^a^Absolute number or mean ± SD/range is provided unless specified.

^b^Delivered in a single fraction.

^c^Excluding repeat SBRT performed in one patient.

^d^1 patient had acute suppression of sustained VT during SBRT.

^e^To avoid toxic dose to stomach.

^f^Three patients underwent two SBRT procedures (36 patients and 39 procedures).

^g^Out of 36 in extended median follow-up 26.9 months for safety cohort.

^h^Bolded row provides summary of the findings from the included studies.

^i^Excluding not available.

^j^The denominator includes safety cohort patients from Hašková *et al*.

### Adverse events

Out of 225 patients, the most common major adverse events were lung- (pneumonia/pneumonitis/pulmonary fibrosis, 13 events or 5.8%) and pericardial-related (pericarditis/pericardial effusion/fibrosis/fistula, 18 events or 8.0%) complications (*Table 2*). These included one reported incident of late gastropericardial fistula and one late oesophago-pericardial fistula.^12,33^ In total, 36% of the patients died during reported follow-up.

### Ventricular tachycardia event rates

The number of cumulative total VT events and patient-months accrued pre- and post-SBRT is shown in *Table 3* (without any imputed missing values). Pre-SBRT, there were 1144 patient-months of data (overall rates of VT episodes 25.7, ATP therapies 26.9, and ICD shocks 2.0 per patient-month). After post-SBRT blanking period, there were 1732 patient-months of follow-up (overall rates of VT episodes 2.3, ATP therapies 3.6, and ICD shocks 0.3 per patient-month).

**Table 3:** Tabulation of cumulative number of clinical VT outcome events prior to and after (excluding blanking period) cardiac SBRT among all patients as reported in the included studies^a^

Number	First author, year	Pre-SBRT					Post-SBRT (after 6-wk blanking)					Blanking period used for analysis
		*n*	VT episodes^b^	ATP therapies^b^	ICD shocks	Patient-months	*n*	VT episodes^b^	ATP therapies^b^	ICD shocks	Patient-months	
1	Cuculich P. 2017	5	6577	6522	55	15	4	4	3	1	46	6 weeks
2	Robinson C. 2019	17	1778	–	292	96	16	111	–	29	72	6 weeks
3	Neuwirth R. 2019^c^	10	212	–	–	30	10	201	–	–	252	3 months
4	Lloyd M. 2020, Wight J. 2022^d,e^	10^f^	852	415	70	24	8	520	418	42	47	None
5	Gianni C. 2020^c^	5	299	201	15	60	5	283	173	64	45	3 months
6	Ho L. 2021	6	91	–	25	21	5	23	–	1	91	6 weeks
7	Yugo D. 2021	3	144	168	15	8	2	3	3	0	24	6 weeks
8	Chin R. 2021^g^	8	591	1233	78	86	7	196	395	36	81	1 month
9	Ho G. 2021	6	–	898	136	36^h^	5	–	72	2	27	6 weeks
10	Carbucicchio C. 2021	7	203	189	11	21	6	74	74	3	20	6 weeks
11	Lee J. 2021	7	332	–	7	42^i^	5	51	–	0	22.5	6 weeks
12	Qian P. 2022^d^	6	1023	–	101	36	6	681	–	2	34.4	None
14	Aras D. 2022^c^	8	3825	3673	203	24	8	95	75	62	42	3 months
15	Ninni S. 2022	17	714	–	–	204	15	138	–	–	212.5	6 weeks
17	Ree M. 2023	6	486	324	87	72	6	154	153	1	57	6 weeks
18	Amino M. 2023	3	143	89	54	18	3	24	6	18	37.5	6 weeks
19	Krug D. 2023	5	353	–	62	15	3	4	–	0	25.5	6 weeks
20	Herrera Siklody C. 2023^d^	20	7419	–	–	120	20	690	–	–	111	None
21	Miszczyk M. 2023^c^	11	394	202	103	30	10	49	30	12	139.5	3 months
22	Arkles J. 2024	14	461	418	44	84	12	48	14	0	112	6 weeks
23	Hašková J. 2024^d^	17	–	1244	255	102	17	–	1884	117	233	None
**21 studies^j^**	**Total**	**191**	**25 897**	**15 576**	**1613**	**1144**	**173**	**3349**	**3300**	**390**	**1732**	
	**Episodes/pt-mo**		**25.7**	**26.9**	**2.0**			**2.3**	**3.6**	**0.3**		

^a^Total number of events among all study patients.

^b^Definitions for counting of VT episodes and ATP therapies differed in different studies.

^c^Three-month instead of 6-week blanking period.

^d^No blanking period.

^e^This study reported VT seconds not VT episodes; VT episodes assumed to be VT seconds/30 s.

^f^Data were not available from Wight *et al*. Data for 10 patients included here were published separately (Lloyd *et al.*).

^g^One-month instead of 6-week blanking period.

^h^ *n* = 5 and 30 patient-months for ATP therapies.

^i^Thirty patient-months for VT episodes.

^j^Bolded rows provide summary of the findings from the included studies.

### Meta-analyses

**Figure 1:**
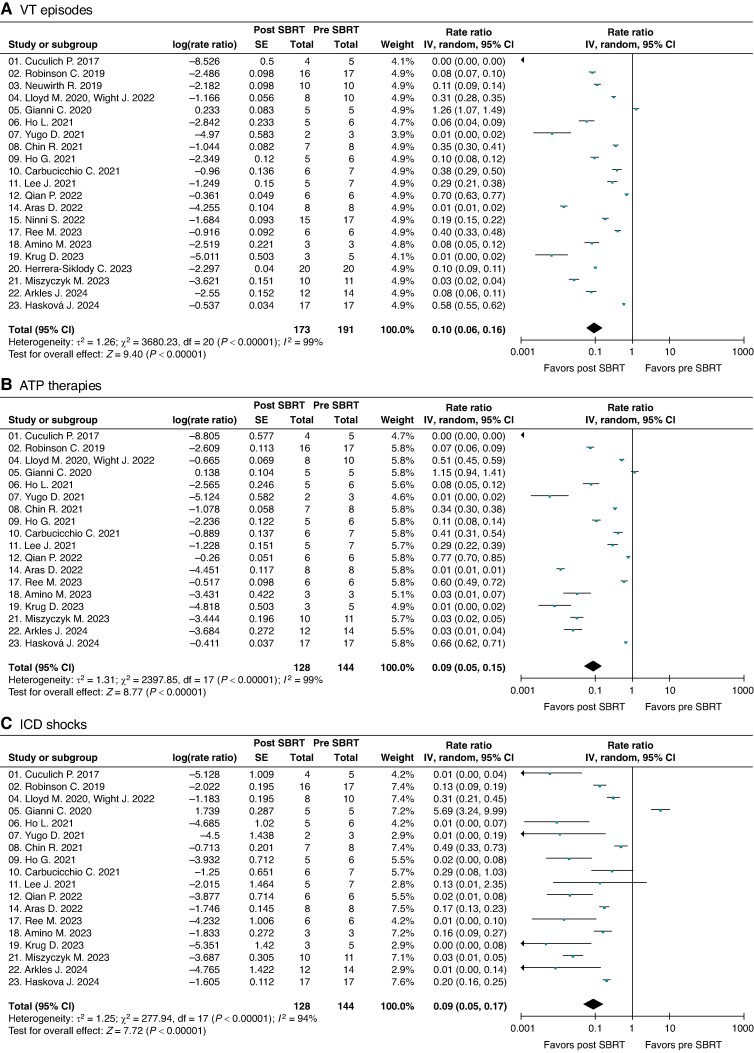
Meta-analyses forest plots depicting the rate-ratios of (*A*) VT episodes, (*B*) ATP therapies, and (*C*) ICD shocks post- (excluding blanking period) vs. pre-SBRT.

Of the 191 patients included in the pooled efficacy VT events meta-analyses, 18 patients died during blanking period and did not contribute post-SBRT VT event data (*n* = 173). There was significant heterogeneity in results from different studies (*χ*2 *P* < 0.00001 for all three endpoints, *I*^2^ VT episodes 99%, ATP therapies 99%, and ICD shocks 94%). The random-effects pooled rate-ratios for VT episodes, ATP therapies and ICD shocks post- (after blanking) vs. pre-SBRT were 0.10 (95% CI 0.06, 0.16), 0.09 (0.05, 0.15), and 0.09 (0.05, 0.17), respectively (all *P* < 0.00001) (*Figure 1*).

Sensitivity analysis by individually removing one study at a time did not materially change any results. Funnel plots to assess for publication bias (see Supplementary material online, *Figure S2*) identified three outlier studies both for VT episodes and ATP therapies that strongly favoured cardiac SBRT (Cuculich *et al*. *n* = 5, Yugo *et al*. *n* = 3, and Krug *et al*. *n* = 5). Sensitivity meta-analyses with exclusion of these outlier studies for all three efficacy outcomes marginally increased the rate-ratio point-estimates for VT episodes to 0.16 (0.10–0.27), ATP therapies to 0.17 (0.10–0.29), and ICD shocks to 0.12 (0.07–0.23), all *P* < 0.00001 (see Supplementary material online, *Figure S3*).

Sub-analyses of the main meta-analyses for overall VT episodes and ATP therapies performed with using as reported events without imputing the missing values did not meaningfully change the results (see Supplementary material online, *Figure S4*).

### Cardiac function

The mean baseline left ventricular ejection fraction reported in the studies ranged 20–45% with 16 out of 23 studies having a mean ejection fraction <35%. The post-SBRT left ventricular ejection fraction was inconsistently reported, with some studies reporting an improvement with others reporting no significant change. As shown in *Table 4*, the overall combined pre-SBRT ejection fraction was 30.9 ± 12.9% and that reported from post-SBRT follow-up was 32.4 ± 13.1%, with average improvement of 1.5% (*P* = 0.3).

**Table 4:** Left ventricular ejection fraction prior to and during follow-up after cardiac SBRT reported in the studies

Number	First author, year	*n*	Left ventricular ejection fraction (%)				
			Pre-SBRT		Follow-up post-SBRT		
			Median (range)	Mean ± SD	Median (range)	Mean ± SD	Mean difference
1	Cuculich P. 2017	5	(15–37)	23 ± 9	(change −2 to +22)	29	+6
2	Robinson C. 2019	19	25 (15–58)	29 ± 12^a^	N/A^b^		–
3	Neuwirth R. 2019	10	(20–35)	27 ± 3	No change	27 ± 3^c^	+0
4	Lloyd M. 2020 (Wight J.2022)	10 (14)	N/A		N/A		–
5	Gianni C. 2020	5	25 (20–55)	34 ± 15	No change	34 ± 15^c^	0
6	Ho L. 2021	7	43 (20–69)	45 ± 18	42 (22–72)	48 ± 20	+3
7	Yugo D. 2021	3	(20–59)	41 ± 20	No change	41 ± 20^c^	0
8	Chin R. 2021	8	20 (15–33)	21 ± 7	N/A	27 ± 4	+6
9	Ho G. 2021	6	26 (10–46)	29 ± 13	N/A		–
10	Carbucicchio C. 2021	7	21 (20–44)	27 ± 11	(*n* = 4)	32 ± 5	+5
11	Lee J. 2021	7	(15–45)	27 ± 10	No change (*n* = 5)	27 ± 10^c^	0
12	Qian P. 2022	6	20 (IQR 16–20)	18 ± 4^a^	20 (IQR 13–20)	17 ± 7^a^	0
13	Molon G. 2022	6	(20–42)	29 ± 9	N/A		–
14	Aras D. 2022	8	25 (10–30)	24 ± 5	N/A		–
15	Ninni S. 2022	17	35 (20–53)	34 ± 10	N/A	35 ± 11	0
16	Chang W. 2022	5	32 (24–57)	34 ± 12	N/A	39 ± 12	+5
17	van der Ree M. 2023	6	38 (24–52)	38 ± 10	N/A	36 ± 6	−2
18	Amino M. 2023	3	(20–65)	37 ± 24	N/A		–
19	Krug D. 2023	5	(20–45)	35 ± 9	(30–60)	43 ± 11	+8
20	Herrera Siklody C. 2023	20	31 (20–72)	37 ± 15	N/A		–
21	Miszczyk M. 2023	11	27 (20–40)	28 ± 7	28 (15–57)	32 ± 14	+4
22	Arkles J. 2024	14	N/A	32 ± 15	N/A	32 ± 12	0
23	Hašková J. 2024	36	N/A	31 ± 10	N/A	31 ± 10	0
**Median (range) for studies**			**26 (20–43)**	**30 (18–45)**	**26.5 (20–42)**	**32 (17–48)**	
**Patient summary**	**Weighted average**			**30.9** ± **12.9**		**32.4** ± **13.1**	**+1.5^d^**

^a^Imputed.

^b^In two patients LVEF improved by 13% and 8%.

^c^Assumed same as baseline based on reported as ‘no change’.

^d^ *P* = 0.3 (independent sample *t*-test).

### Time-to-event analyses

**Figure 2:**
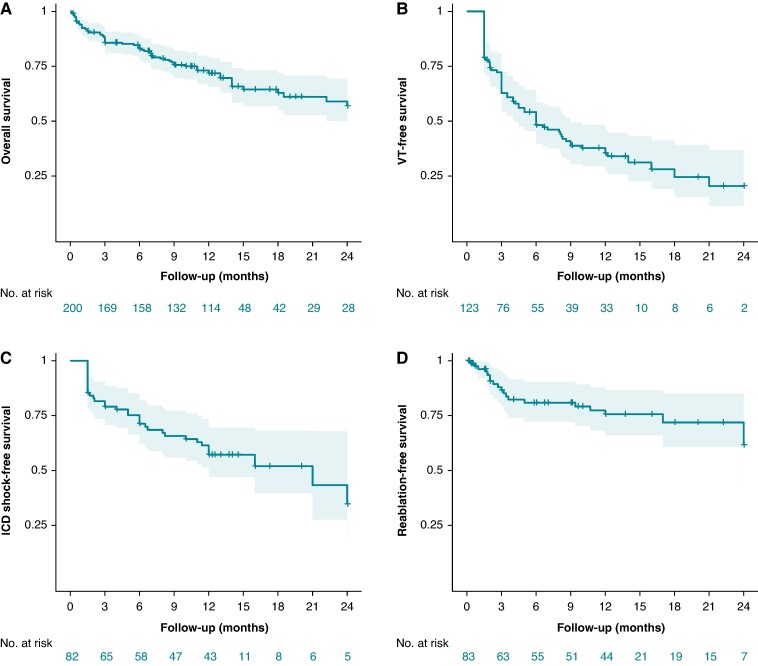
Kaplan–Meier curves with 95% CI depicting (*A*) overall survival, (*B*) recurrent VT (post-blanking)-free survival, (*C*) ICD shock (post-blanking)-free survival, and (*D*) redo catheter ablation/SBRT-free survival.

KM analyses of the study population showed 3-, 12-, and 24-month overall patient survival 0.86 (0.80, 0.90), 0.72 (0.65, 0.78), and 0.57 (0.47, 0.67), respectively. The other time-to-event analyses were limited by missing data. However, based on the available data, at 6 months post-SBRT, the rate of survival without any VT recurrence (after blanking) was 0.47 (0.38, 0.56), ICD shock-free survival (after blanking) was 0.71 (0.60, 0.80), and repeat catheter ablation/SBRT-free survival was 0.81 (0.70, 0.89) (*Figure 2*). The summary of time-to-event analysis for individual studies is available in Supplementary material online, *Table S2A*–*C*.

## Discussion

Refractory VT in setting of structural heart disease, often advanced heart failure, presents a clinical challenge due to limited therapeutic options and high mortality. Early reports of clinical experience with SBRT for refractory VT has led to its enthusiastic adoption across multiple centres. We performed a comprehensive pooled analysis of data from published studies on cardiac SBRT. This is important given the lack of randomized controlled trials on this topic.

### Salient findings

**Figure 3:**
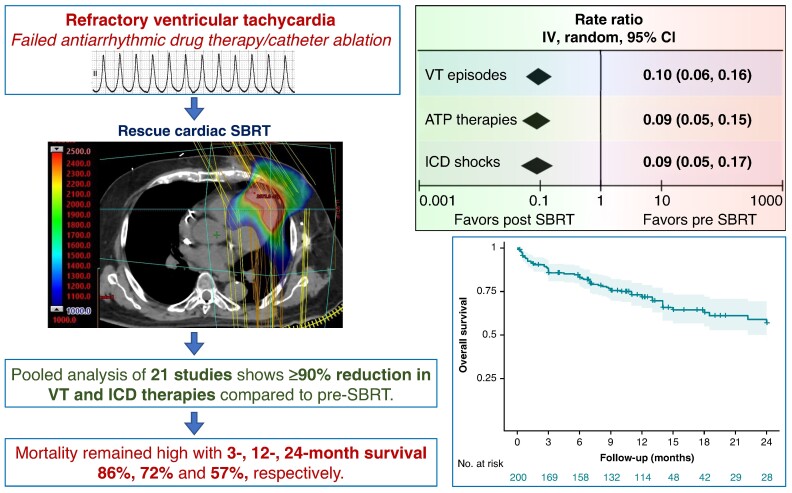
Schematic representation of the effect of cardiac SBRT on VT outcomes and post-treatment overall survival. VT, ventricular tachycardia; ATP, anti-tachycardia pacing; ICD, implantable cardioverter-defibrillator; SBRT, stereotactic body radiotherapy.

This is to-date the largest systematic review on outcomes with cardiac SBRT for refractory VT, encompassing a total of 23 studies and over 200 patients. There was considerable heterogeneity in the indications for treatment, procedure characteristics, follow-ups, and outcomes among the included studies. The salient findings, as summarized in *Figure 3*, are as follows. First, among patients with refractory VT, there was on average a 10-fold reduction in the rate of VT episodes and ICD therapies/shocks after cardiac SBRT. Secondly, most complications with cardiac SBRT were minor and mostly comprised of radiation-related changes in the pericardium, lungs and oesophagus/stomach. However, severe complications, i.e. oesophagogastro-pericardial fistulas, though uncommon, can occur months to years after treatment and require long-term follow-up and high index of suspicion for early detection to prevent fatality. Though some patients had progression of mitral regurgitation, there was no suggestion of a systematic decline in cardiac function following SBRT. Thirdly, somewhat expectedly, this population comprising of patients with advanced heart failure and refractory VT having failed antiarrhythmic drugs and catheter ablation had increased mortality despite cardiac SBRT (43% over 2 years).

### Magnitude of benefit

As described previously, ∼10-fold reduction in VT episodes, ICD therapies and shocks was noted in our study. However, the magnitude of benefit was not uniform across studies. Nevertheless, a relatively consistent reduction in ICD shocks was seen, suggesting an improved quality of life which is important when considering cardiac SBRT with palliative intent. Despite a significant reduction in VT-related events, complete remission of VT episodes was uncommon, with more than half the surviving patients having VT recurrences and almost 30% of receiving ICD shocks within 6 months post-SBRT. From the few studies that reported this outcome, a fifth of the surviving patients had to undergo reablation by 6 months of the index SBRT procedure, though there was heterogeneity in threshold for repeat ablation in different studies.

### Comparison with catheter ablation

Catheter ablation is the standard of care for management of drug refractory VT.^8,36^ There have been no head-to-head studies comparing SBRT with catheter ablation. Virk and Kumar^37^ conducted a meta-analysis comparing catheter ablation with medical therapy alone, which showed an 18% lower VT recurrence, and a 38% lower rate of ICD shocks in the catheter ablation group. In our analysis, we observed a 90% reduction in VT recurrence and a 91% reduction in ICD shocks after SBRT. While these results may appear impressive vis-à-vis catheter ablation trials, it is important to note that there are differences in included study populations, and this is a temporal comparison without a true control group and is susceptible to survivorship bias and regression-to-the-mean. Consequently, randomized controlled trials comparing SBRT with catheter ablation are required to make valid comparisons between the two modalities. RADIATE-VT (NCT05765175) is an ongoing international, multicentre, randomized controlled trial comparing SBRT to catheter ablation in patients with high-risk refractory VT that is expected to be completed in 2026.

Stereotactic body radiotherapy has some logistical advantages over catheter ablation. It is non-invasive and can be tolerated by patients with poor hemodynamics that would otherwise prohibit a catheter ablation procedure under general anaesthesia, although electrophysiology study with VT induction for non-invasive mapping using electrocardiographic imaging or invasive mapping may still be required to identify the target. Additionally, delineating the VT substrate for SBRT often requires extensive non-invasive work-up that may include cardiac contrast computed tomography (CT), myocardial positron emission tomography (PET) perfusion scan, and/or cardiac magnetic resonance imaging (MRI). As exemplified by case reports (see Supplementary material online, *Table S1A* and *B*) SBRT can be offered despite left ventricular thrombus or mechanical valves that may preclude endocardial catheter access, and pericardial fibrosis that may limit epicardial access. Additionally, catheter ablation may be unable to access mid-myocardial fibrosis particularly in setting of myocardial hypertrophy.

### Adverse events

The heart is surrounded by several radiation susceptible tissues, including the gastrointestinal tract, lungs, and pericardium. Radiation-induced injury to actively dividing epithelial cells in these tissues can lead to potentially serious adverse events. While pericardial-related and lung-related events were commonly observed after SBRT, these were either transient or had limited effects on the functional status of patients. Further, there have been multiple reports of gradually developing, often asymptomatic pericardial effusions following SBRT although none reported pericardial tamponade. Nevertheless, the index of suspicion should stay high for this potentially fatal complication. Oesophagogastro-pericardial fistulas can be a devastating and often fatal late-complication. We found two reports, one oesophago-pericardial and one gastropericardial fistula.^38,39^ Oesophago-pericardial fistula was reported in a 67-year-old male who had a prior history of coronary artery revascularization using a graft from the gastroepiploic artery and developed radiation-induced oesophagitis 18 days post-SBRT treated with antiulcer therapy. However, he presented with an oesophageal ulcer 6 months post-SBRT and eventually died due to bleeding from the atrioesophageal fistula 9 months post-SBRT. The patient with gastropericaridal fistula presented 2 years post-SBRT, underwent emergent surgical repair and survived. Therefore, caution is warranted when planning targets in proximity to luminal structures. Techniques like deep inspiration breath-hold can be employed to mitigate such complications by reducing the radiation dose to gastrointestinal tract.^40^

### Structural cardiac effects

Conventional radiotherapy for treatment of chest malignancies has been associated with increased risk of various cardiac diseases, including coronary artery disease, valvulopathy, pericarditis, cardiomyopathy, and conduction abnormalities.^41^ With a higher cardiac radiation dose, adverse cardiac effects may be even more prominent with SBRT for VT. Notably, many of the radiation-related adverse effects on cardiac function develop over years to decades, assuming less of an importance in this patient population with reduced survival. Reassuringly, we did not find any signal of a decline in left ventricular systolic function with cardiac SBRT (average ejection fraction 30.9% pre-SBRT to 32.4% during follow-up), with the caveats of short duration of follow-up and survivorship bias. Several studies reported heart failure exacerbation as an adverse event, although only two events had a possible attribution to SBRT.^12^ Progression of mitral regurgitation was seen in some studies, developing over a period of 6 months to 2 years. Hašková *et al*.^33^ reported eight cases out of 36 with progression of mitral regurgitation and found that irradiation of basal inferior-to-lateral segments was associated with increased risk of progression. These patients developed posterior leaflet restriction, hinting at radiation-induced fibrotic changes within the valvular apparatus. There is concern for thromboembolic events following cardiac SBRT, with one patient in the seminal case series by Cuculich *et al*.^11^ suffering a fatal stoke and van der Ree *et al*.^27^ reporting an intracardiac thrombus. As a result, most centres anticoagulate patients for up to 3 months post-cardiac SBRT. Reassuringly, no coronary events were reported in any of the included studies.

### Antiarrhythmic mechanism

SBRT kills cancer cells directly by causing double-strand DNA breaks, and indirectly by disrupting vasculature and stimulation of anti-tumour immune responses.^42^ Contrary to actively dividing cancer cells, which possess open chromatin and replicating DNA susceptible to radiation injury, cardiomyocytes remain quiescent with tightly packed DNA. Therefore, the antiarrhythmic mechanisms of cardiac SBRT differs significantly from cancer ablation.^43^ Animal studies have shown radiation-induced transmural fibrosis can account for antiarrhythmic effects of cardiac SBRT.^44^ However, radiation-induced fibrosis of the pulmonary vein myocardium takes months to develop and requires higher radiation doses (>30 Gy). This contrasts with the immediate clinical response seen with ventricular SBRT for VT even at a lower dose of 25 Gy. In animal models, the cardiac proteome exhibits dynamic alterations in various domains following SBRT, notably conduction proteins.^45^ Therefore, the more probable mechanisms involve electrical reprogramming, characterized by heightened expression of Nav1.5 and Cx43 channels, increasing conduction and reducing dispersion in repolarization.^46^ Other physiological responses to radiation, including inflammation and microcapillary thrombosis may contribute to the overall treatment response as well.^43,47^ The notion of traditionally described physiological responses to SBRT, including resolution of inflammation and development of fibrosis over weeks to months, has been the basis for selecting blanking periods ranging from 6 weeks to 3 months. However, owing to the immediate clinical response seen with SBRT in the context of VTs, and the growing *in-vitro* literature which supports electrical reprogramming as one of the mechanisms for clinical response, many clinical studies have questioned or removed the blanking period altogether.^22,30^ The 2024 consensus statement by European Heart Rhythm Association (EHRA) and Heart Rhythm Society (HRS) on SBRT for VT recommends reporting all VT recurrences regardless of blanking period.^48^ The reported experience on cardiac SBRT is heavily dominated by dose of 25 Gy delivered in a single fraction, though there are reports of successful cases with lower dose or the dose divided in multiple fractions (see Supplementary material online, *Table S1A*). Beyond refractory VT, as shown in Supplementary material online, *Table S1A* case reports, there is proof-of-concept demonstrating SBRT as treatment of ventricular fibrillation, treatment of intracardiac tumours, and targeting VT in setting of hypertrophic, arrhythmogenic, and Chagas cardiomyopathies.

### Mortality

Patients experiencing a high VT burden resistant to catheter ablation constitute a vulnerable cohort with elevated mortality. The 1-year mortality in our study population of 28% is consistent with 32% reported by Nagashima *et al*.^49^ in those with early recurrence of VT following catheter ablation. Recurrent VT may be both a mechanism of mortality as well a marker of progressive heart failure that is a harbinger of death. Interestingly, in the VANISH trial, which was the largest randomized controlled trial comparing catheter ablation with escalation of drug therapy in patients experiencing VT on antiarrhythmic drugs, mortality was similar in both groups with a 1-year mortality 10–15%.^8^ Similarly, in the meta-analysis by Virk and Kumar^37^, despite reductions in VTs and ICD shocks with catheter ablation, no difference in mortality was noted. However, when Reddy *et al*.^50^ did a patient-level meta-analysis, catheter ablation was associated with a statistically significant reduction in mortality. Most patients included in our study had already received multiple prior catheter ablations, indicating a population with more advanced disease. In fact, most patients were not deemed candidates for repeat catheter ablation and thus would not have qualified for inclusion in the above stated catheter ablation trials. It is unlikely that SBRT itself was directly related to deaths. In a prior meta-analysis on SBRT for VTs by Benali *et al*.,^51^ 52% of deaths occurring within 1 year of SBRT were attributed to progression of heart failure, and only 6.5% of the deaths were attributed to refractory ventricular arrhythmias. Given the presence of competing causes of mortality in this critically ill population, randomized controlled trials are desired to isolate the effect of cardiac SBRT on mortality outcomes.

### Statistical context

We need to put our results in context of the statistical limitations. First, the studies included in our analysis are small and heterogenous in methodology and results. All studies were uncontrolled without a true comparator group. Temporal comparison of post-SBRT outcomes with events prior to treatment has major drawbacks. Such comparisons in context of a lethal disease like VT which presents in episodic clusters of VT storms are susceptible to the phenomena of regression-to-the-mean and survivorship bias, which can exaggerate the apparent efficacy of cardiac SBRT. Further, the implicit assumption of a static risk over time in the calculation of risk ratios is not accurate.

### Other limitations

First, the included studies were heterogenous with differences in patient selection, antiarrhythmic drug therapy, previous VT ablations, SBRT planning/delivery, and reporting of outcomes. Moreover, our efficacy endpoints, i.e. VT episodes and ATP therapies, are influenced by the programming settings of individual ICDs. The lack of uniform programming approaches could have impacted our results. Secondly, the included reports were single arm retrospective or prospective series with small sample sizes, affecting the quality of evidence. Thirdly, there was missing data for many outcomes necessitation manual data extraction from figures and imputation of missing values possibly introducing inaccuracies. Further, this analysis is subject to publication bias and other statistical fallacies with retrospective meta-analyses. Many of the included studies lacked long-term follow-up information, which can lead to underestimation of complications. We note that the reports on cardiac SBRT for VT are heavily dominated by the experience in white males, and there is a need for evaluation of SBRT specifically in women and other racial groups. However, despite these limitations, our study is the most comprehensive summary of the contemporary literature on cardiac SBRT for refractory VT.

### Future directions

The Standardized Treatment and Outcome Platform for Stereotactic Therapy of Re-entrant Tachycardia by a Multidisciplinary (STOPSTORM) consortium has been established in develop a pooled treatment database for evaluating the outcomes of SBRT and standardizing its practice within the European Union.^52^ A consensus statement has been proposed by EHRA/HRS on SBRT for VT, advising on patient selection, VT substrate delineation reporting of outcomes, and standardization of follow-up.^48^ This statement also emphasizes the need for evaluation of long-term complications associated with SBRT in future studies.

Further, while randomized controlled trials are desired to accurately evaluate the efficacy and safety of cardiac SBRT vis-à-vis catheter ablation, most patients reported in this analysis were end-stage VTs who had failed or were not candidates for catheter ablation and therefore there is no possibility of comparison with catheter ablation.

## Conclusions

Cardiac SBRT is a promising treatment option for patients with VT refractory to catheter ablation and antiarrhythmic drug therapy. We found a significant decrease in VT episodes, ATP therapies, and ICD shocks post-SBRT; however, this high-risk population remained at high risk for mortality. In the absence of randomized controlled trials, the objective efficacy of cardiac SBRT and its effect on long-term outcomes and mortality remains uncertain.

## Supplementary Material

euae305_Supplementary_Data

## Data Availability

The data that support the findings of this study is derived from publicly available peer-reviewed articles, which can be accessed from individual journal websites.
