# Supplementary material for: Stereotactic cardiac radiotherapy for refractory ventricular tachycardia in structural heart disease patients: a systematic review
Source: Europace. 2024 Dec 24;27(1):euae305. doi: 10.1093/europace/euae305 (PMC11780863; doi:10.1093/europace/euae305)
Supplement: euae305_Supplementary_Data [file euae305_supplementary_data.docx]

**SUPPLEMENTARY FILE**

**Search Terms (executed on PubMed on March 30^th^, 2024)**

("tachycardia"[MeSH Terms] OR "tachycardia"[All Fields] OR "tachycardias"[All Fields] OR "tachycardia s"[All Fields] OR ("arrhythmia s"[All Fields] OR "arrhythmias, cardiac"[MeSH Terms] OR ("arrhythmias"[All Fields] AND "cardiac"[All Fields]) OR "cardiac arrhythmias"[All Fields] OR "arrhythmia"[All Fields] OR "arrhythmias"[All Fields])) AND ("heart ventricles"[MeSH Terms] OR ("heart"[All Fields] AND "ventricles"[All Fields]) OR "heart ventricles"[All Fields] OR "ventricular"[All Fields]) AND ("radiotherapy"[MeSH Terms] OR "radiotherapy"[All Fields] OR "radiotherapies"[All Fields] OR "radiotherapy"[MeSH Subheading] OR "radiotherapy s"[All Fields] OR ("radiosurgeries"[All Fields] OR "radiosurgery"[MeSH Terms] OR "radiosurgery"[All Fields]) OR ("stereotactic"[All Fields]) OR ("radiate"[All Fields] OR "radiated"[All Fields] OR "radiation"[MeSH Terms] OR "radiation"[All Fields] OR "radiation s"[All Fields]) OR "SABR"[All Fields] OR "SBRT"[All Fields] OR OR "star"[All Fields])

**Google Scholar Search:** Search terms "stereotactic body radiotherapy" "ventricular tachycardia" were executed on March 30^th^, 2024, followed by manual review of first 200 results.

**Review of search results:** The search results were independently reviewed by two authors (AG and ZS), and any conflicts were resolved by one author (AN).

**Data extraction for time-to-event and event burden analysis**

Individual patient-level data for time-to-event and event burden analysis was not explicitly available in the text for some studies. In these cases, we captured data from figures and graphs. We used WebPlotDigitizer v4.7 (Rohatgi 2024), an open-source software for manual data extraction from images. The reporting intervals were heterogenous for the studies, ranging from the exact number of days to 3-month intervals for time to event. In case of data reported as intervals (for example 0-1 months, 1-2 months), the upper limit of the interval was taken as the time to event. Further, in cases where the exact follow up was difficult to estimate, the mean/median follow-up duration was used to censor data. For patients who continued to have VT recurrences/ICD shocks during and after the blanking period, the time to event was taken as 1.5 months (~6 weeks blanking) for imputing in the Kaplan-Meier curves. In cases where VT recurrence data was unavailable, treated VT episodes (ATP + ICD shocks) were taken as proxies. In the study by Hašková et al., all remaining patients had VT recurrence by 12 months and ATP therapies were taken as a proxy for time to recurrence.

**Supplementary Figure 1.** PRISMA flow diagram of literature review and studies included in the meta-analysis.

Records screened

(n = 1157)

Records excluded

(n = 1131)

Reports sought for retrieval

(n = 26)

Reports not retrieved

(n = 0)

Reports assessed for eligibility

(n = 26)

Reports excluded: 0

Studies included in review

(n = 23)

Reports of included studies

(n = 26 including 3 repeat reports)

Records removed *before screening*:

Duplicate records removed (n = 21)

Records marked as ineligible by automation tools (n = 0)

Records removed for other reasons (n = 0)

Records identified from: 1178

Databases (n = 1): 1157Databases

(n = 1 PubMed): 1157

Registers (n = 1): 21Registers

(n = 1 clinicaltrials.gov): 21

**Identification of studies via databases and registers**

**Identification**

**Screening**

**Included**

**Supplementary Figure 2.** Funnel plots for assessing publication bias in VT events post SBRT (excluding blanking period) versus pre SBRT. Dotted red ovoid shows the 3 outlies studies.

1. VT episodes


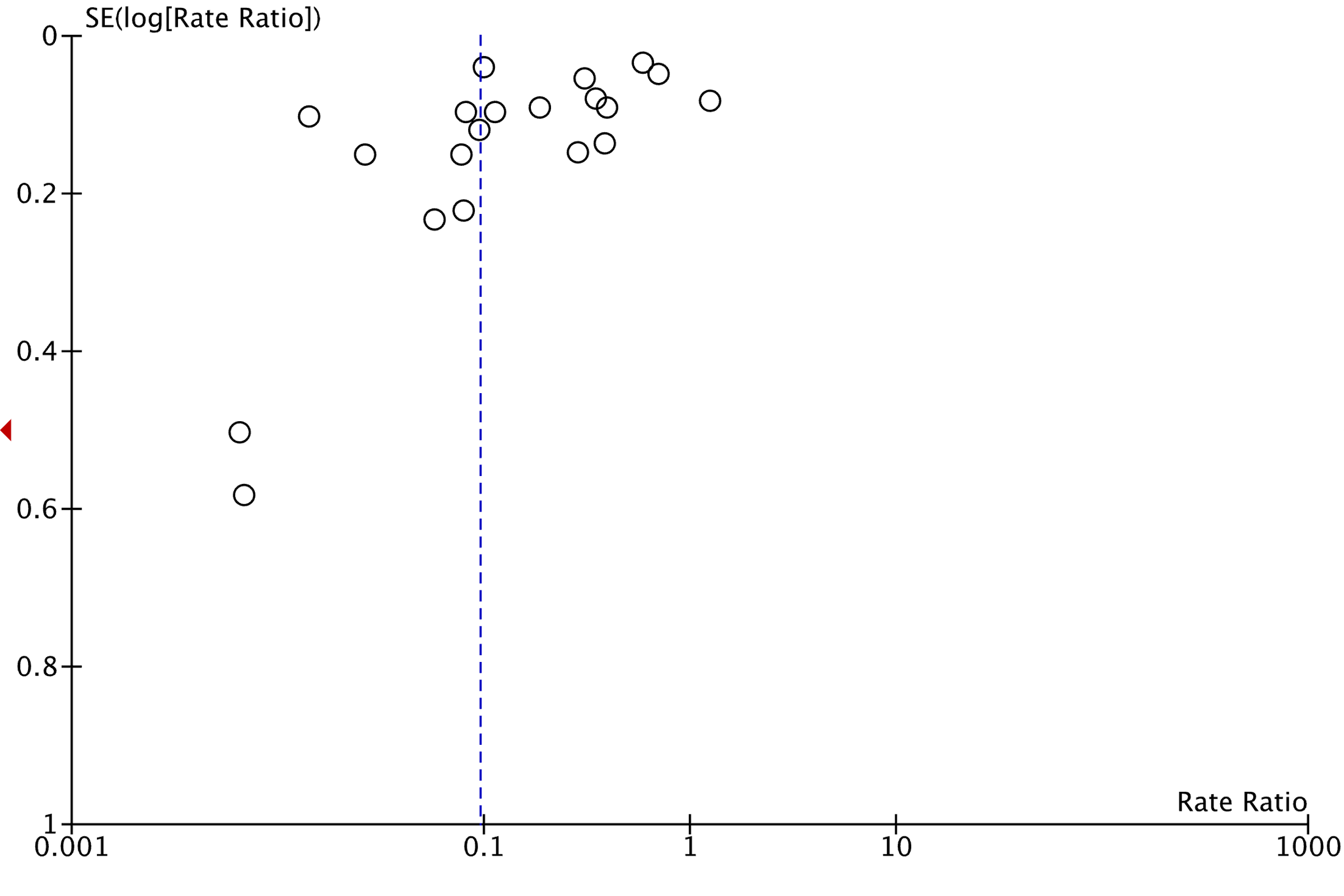


1. ATP therapies


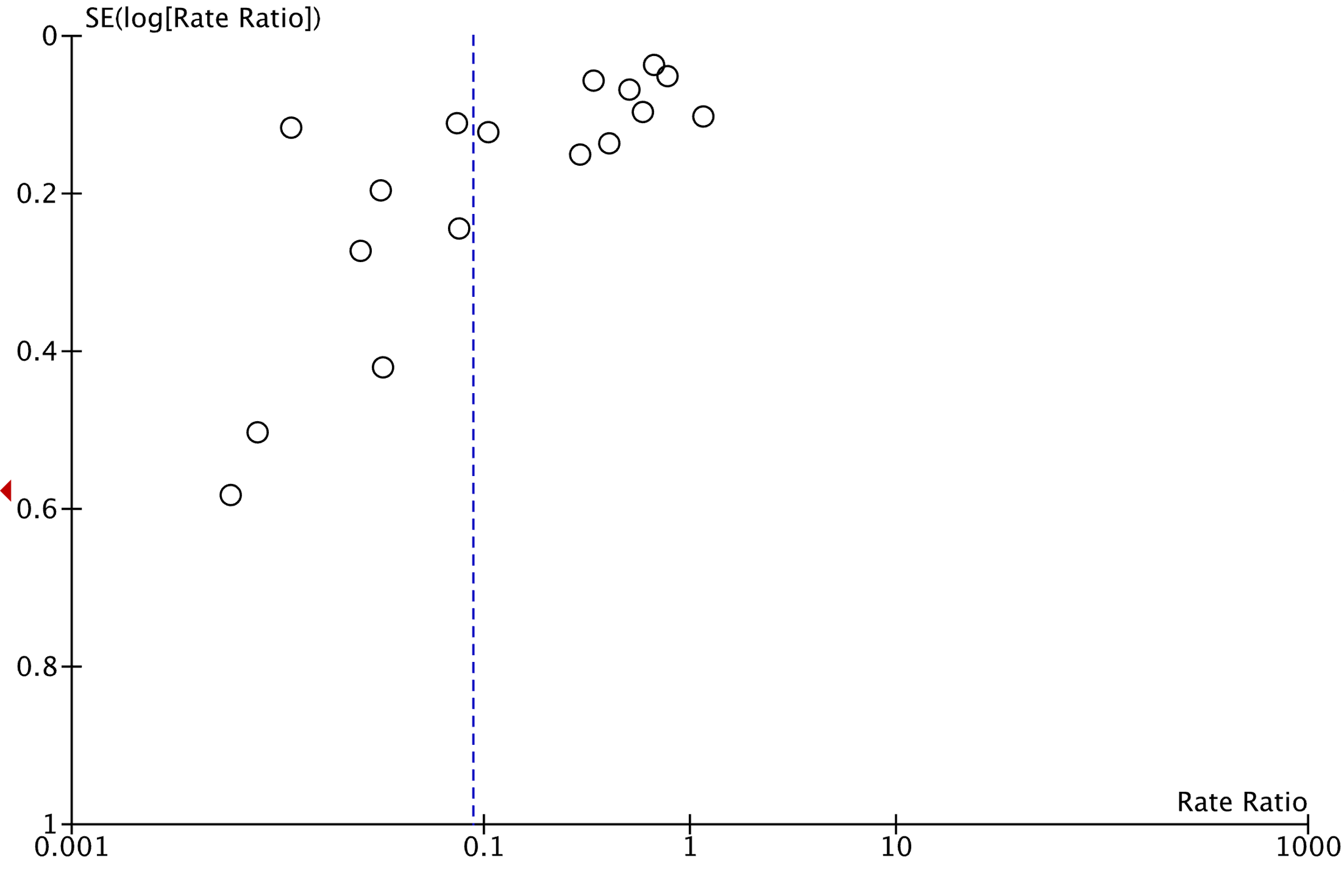


1.
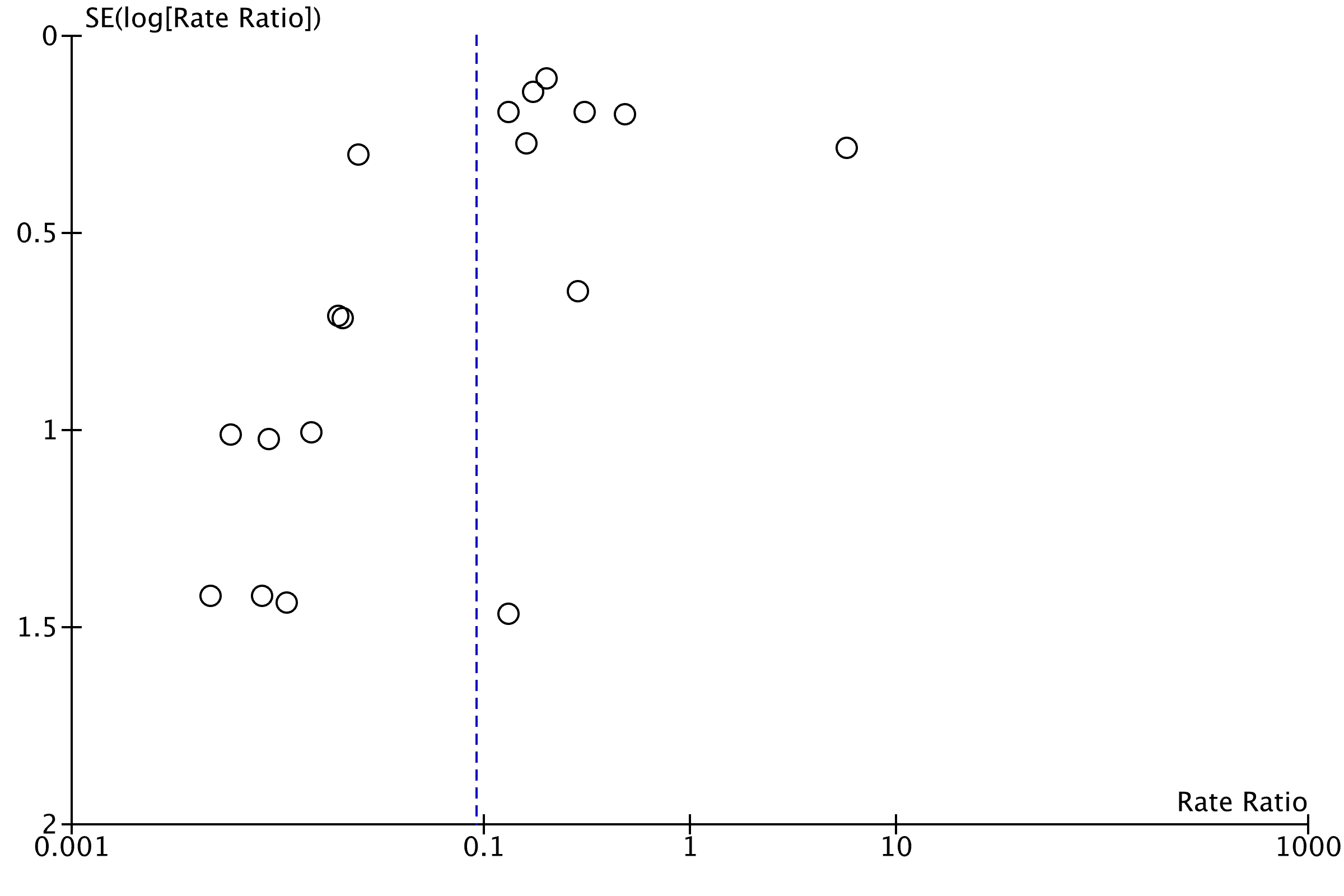
ICD shocks

**Supplementary Figure 3.** Sensitivity analyses forest plots depicting the rate-ratios of (A) VT episodes, (B) ATP therapies, (C) ICD shocks post versus pre SBRT after removing studies reporting outlier results.

- - 1. VT episodes


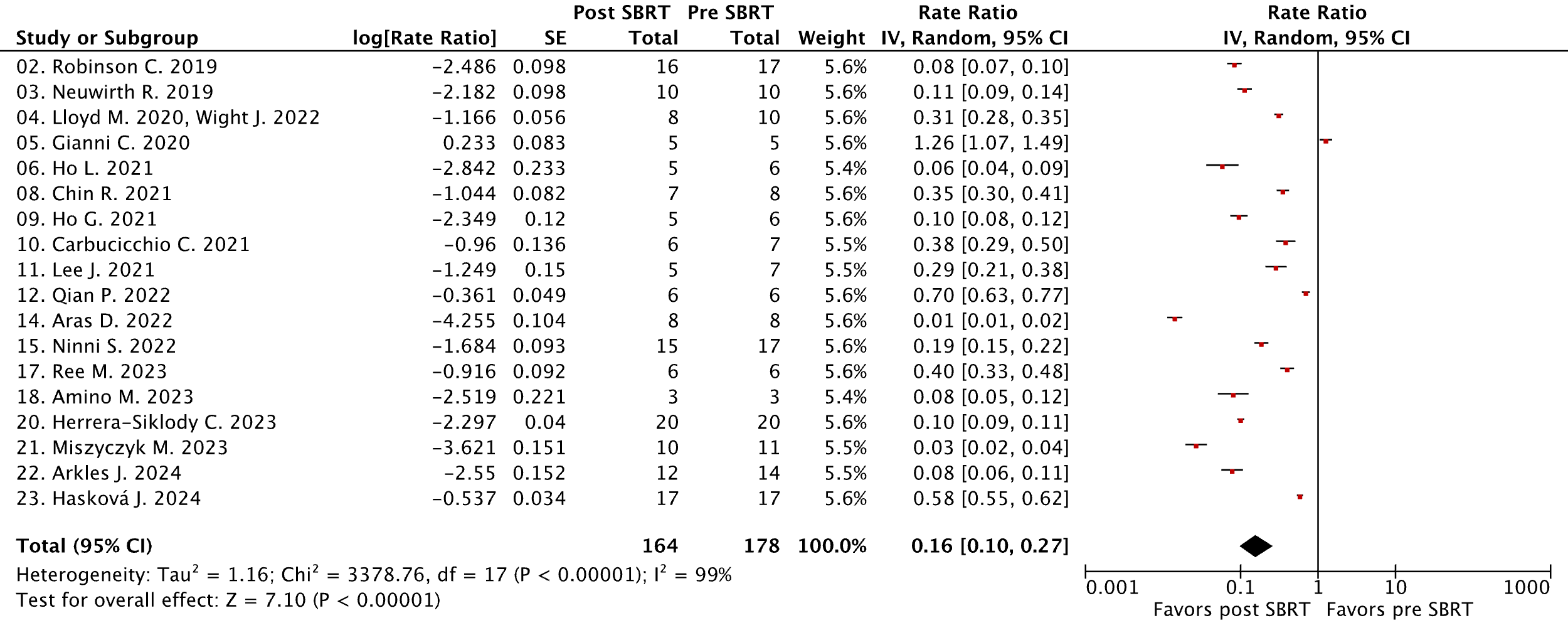


- - 1. ATP therapies


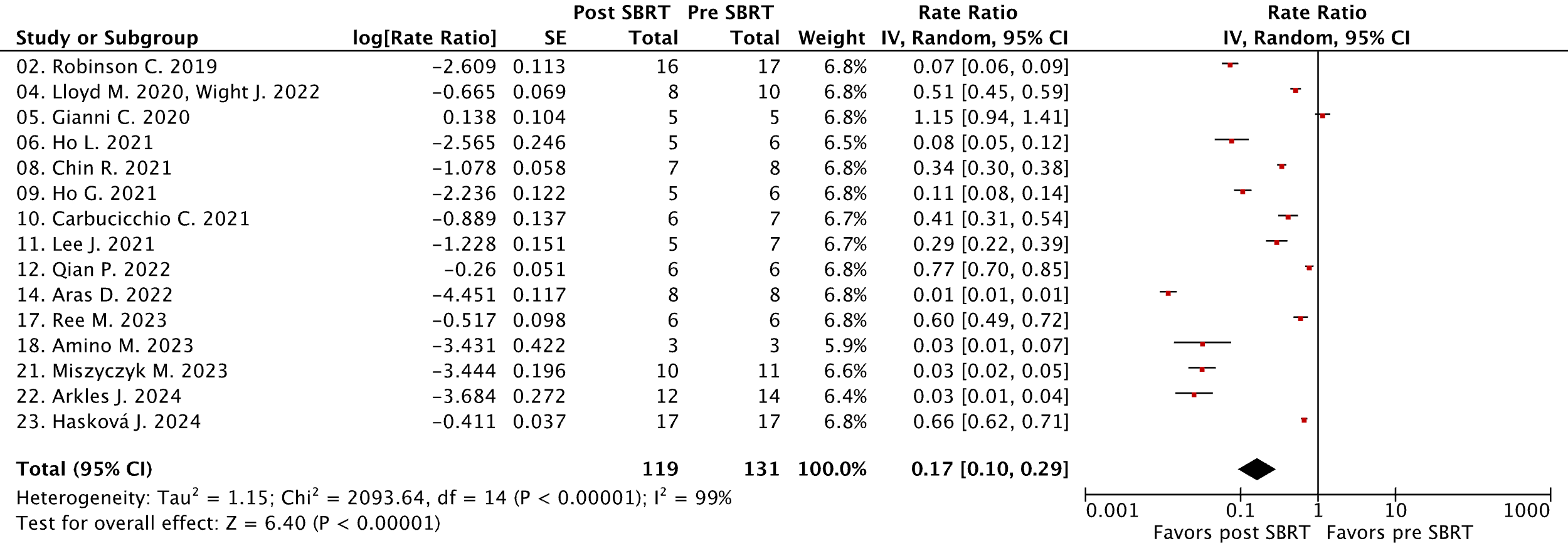


- - 1. ICD shocks


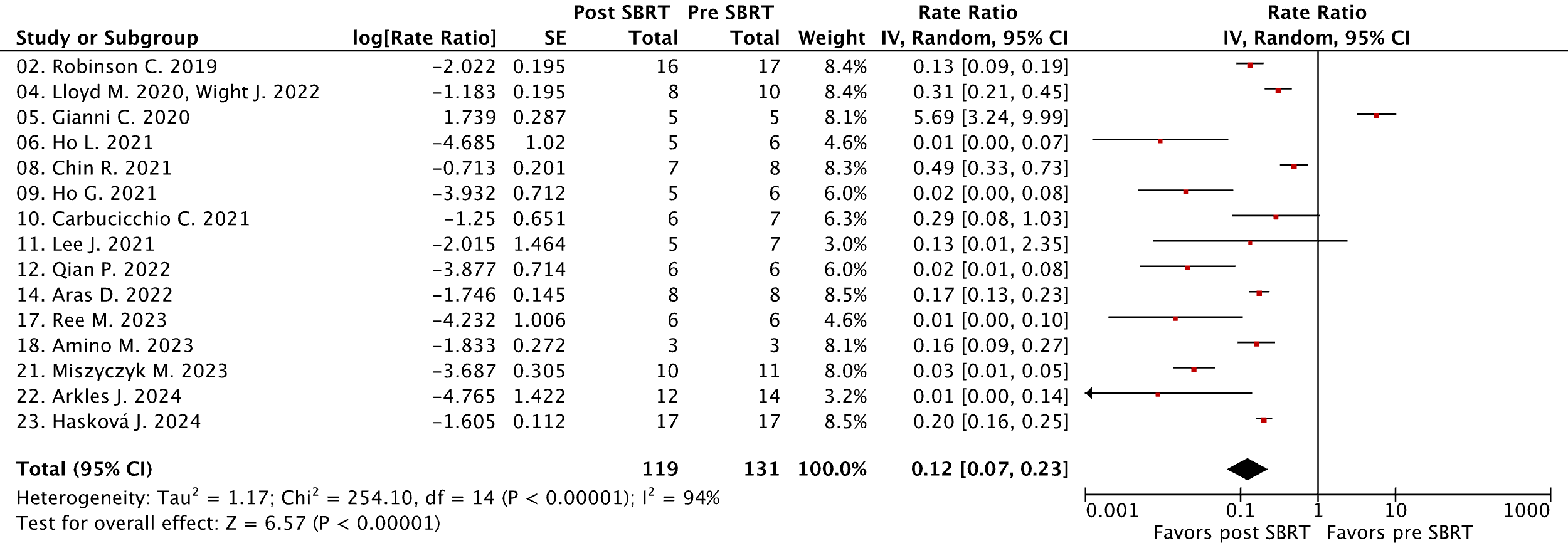


**Supplementary Figure 4.** Meta-analyses forest plots without imputing missing values depicting the rate-ratios of (a) VT episodes and (b) ATP therapies post SBRT (excluding blanking period) versus pre SBRT.

1. VT episodes


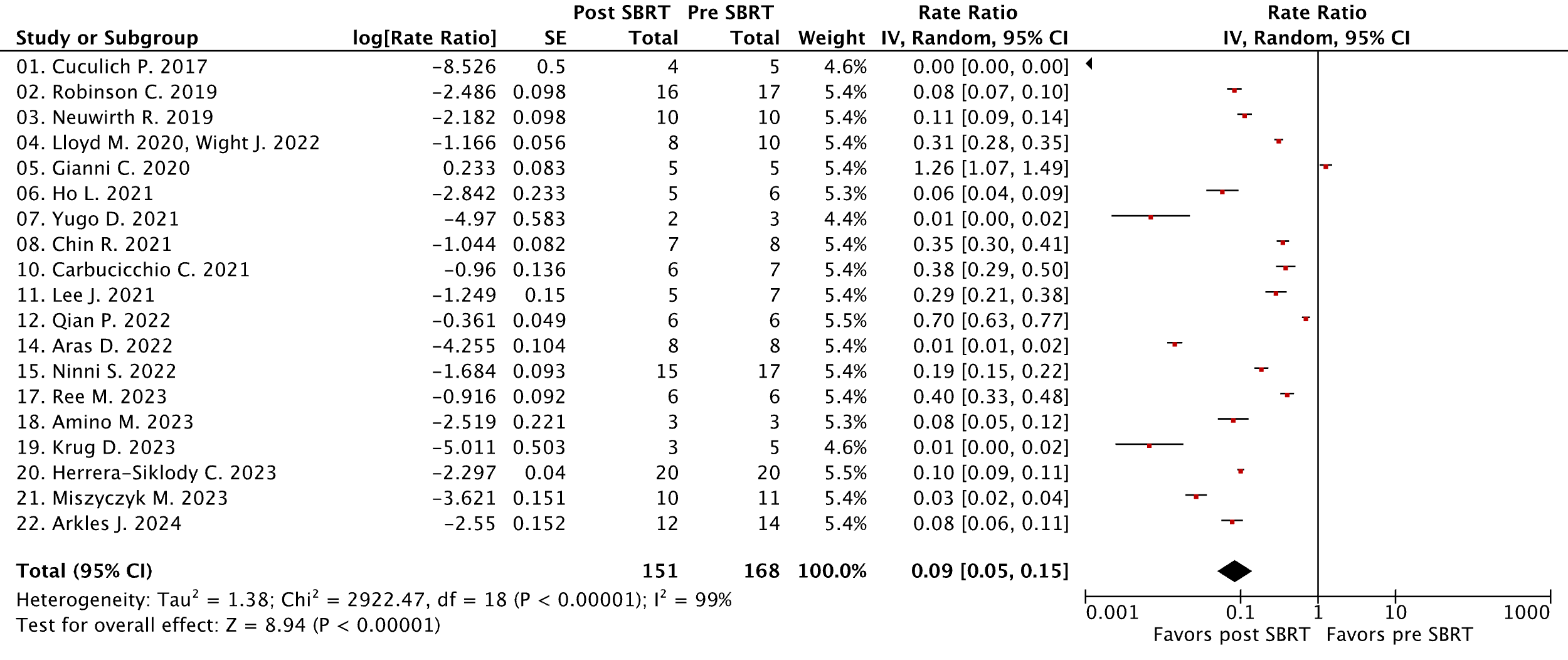


1. ATP therapies


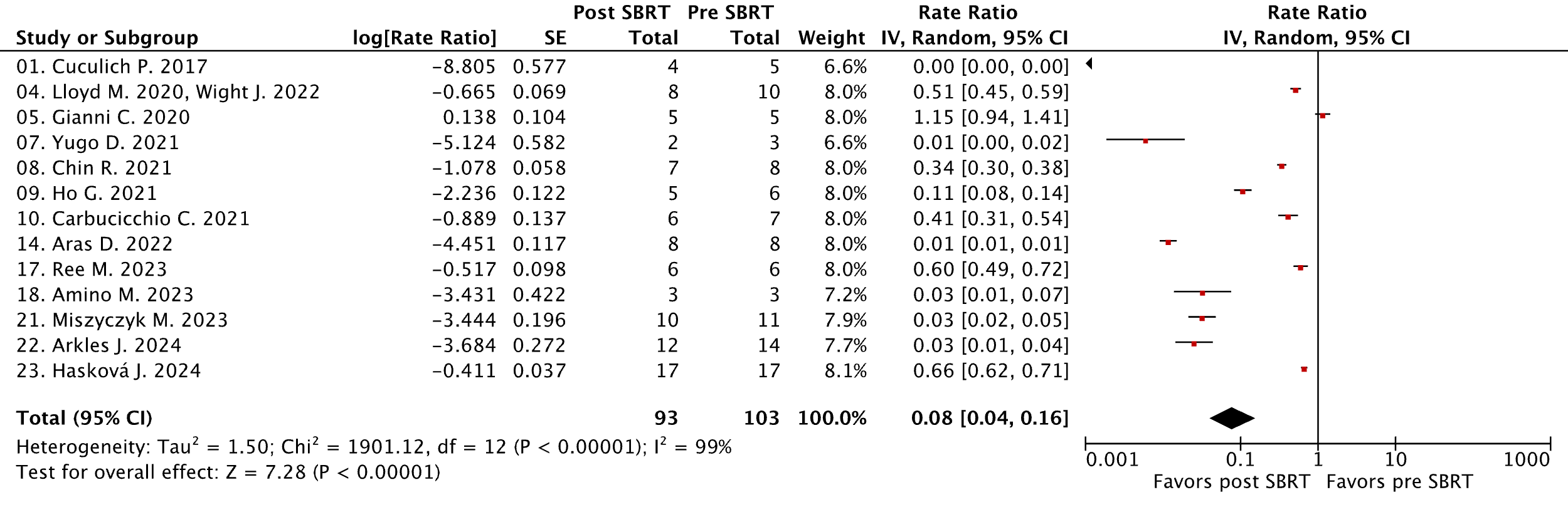


**Supplementary Table 1A**. Summary of case reports on cardiac SBRT for ventricular tachycardia: baseline characteristics.

| **#** | **Author/ Name** | **Publication Date** | **Clinical Presentation** | **Age (years)** | **Sex** | **LVEF** | **Structural Heart Disease** | **Prior VT Therapies** | **RadiationDose** | **Planning Treatment Volume (PTV)** | **Comments** |
| --- | --- | --- | --- | --- | --- | --- | --- | --- | --- | --- | --- |
| 1 | Cvek et al. (1) | Jul 2014 | Refractory VT | 72 | F | 25% | NICM | ADT, CA | 25 Gy | N/A | Initial experience cardiac SBRT for VT |
| 2 | Loo et al. (2) | Jun 2015 | Refractory VT | 71 | M | 24% | ICM | ADT | 25 Gy | N/A |  |
| 3 | Scholz et al. (3) | Mar 2019 | Refractory VF | 53 | M | Recent prox. LAD infarct | ICM | ADT, CA | 25 Gy | 82.4 | SBRT for post-infarct refractory VF |
| 4 | Zeng et al. (4) | Jun 2019 | Refractory VT | 29 | M | 60-75% | LV lipoma | Partial resection of lipoma, ADT, CA | 24 Gy | 71.2 | Lipoma |
| 5 | Bhaskaran et al. (5) | Sep 2019 | Refractory VT | 34 | F | N/A | None | ADT, CA | 25 Gy | 52 | Unrelated metastatic neuroendocrine tumor of paranasal sinus |
| 6 | Hašková et al. (6) | Oct 2018 | Refractory VT | 34 | M | N/A | Intramyocardial benign fibroma | ADT, CA | 25 Gy | N/A | Fibroma |
| 7 | Krug et al. (7) | Oct 2019 | Refractory VT | 78 | M | 15% | Combined ICM/NICM | ADT | 25 Gy | 42.2 | 1^st^ German case |
| 8 | Jumeau et al. (8) | Oct 2019 | Non-sustained VT (non-inducible) | 66 | F | N/A | Intraventricular septal metastatic lung adenoca. | ADT | 19.7-22.3 Gy | 21 | Cardiac metastases related VT |
| 9 | Marti-Almor et al. (9) | Jan 2020 | Refractory VT | 64 | M | N/A | Arrhythmogenic CM | ADT, CA | 25 Gy | 3.5 | Low volume irradiation in RV free wall. |
| 10 | Park et al. (10) | Jul 2020 | Recurrent VT | 76 | M | 63% | Hypertrophic CM | ADT | 24 Gy | N/A | 1^st^ Korean case, 8 Gy x 3 fractions |
| 11 | Narducci et al. (11) | Nov 2020 | Refractory VT | 60 | M | 27% | NICM | ADT, CA | 25 Gy | 303 | - |
| 12 | Fiorentino et al. (12) | Oct 2020 | Recurrent VT | 67 | M | 25% | ICM | ADT | 25 Gy | N/A | 1^st^ Italian case, ^18^F-FDG PET-CT for SBRT planning |
| 13 | Gerard et al. (13) Case 1 | Mar 2021 | Refractory VT | 65 | M | 30% | ICM | ADT, CA | 25 Gy | 102 | - |
| 14 | Gerard et al.  Case 2 | Mar 2021 | Recurrent VT | 65 | M | 35% | ICM | ADT | 25 Gy | 66.4 | CA not done due to LV apical thrombus |
| 15 | Thosani et al. (14) | Apr 2021 | Refractory VT | 73 | M | 45% | NICM | ADT, CA | 25 Gy | 62.6 | - |
| 16 | Hašková et al. (15) Case 1 (16) | Mar 2022 | Refractory VT | 66 | M | 35% | ICM | ADT, CA, SBRT | 25 Gy | 18 | 3 cases of redo cardiac SBRT after 6 weeks incl. Hašková 2018 (6) and Peichl 2020 (16).  Showed that SBRT can be safely repeated in case of recurrence. |
| 17 | Hašková et al. Case 2 | Mar 2022 | Refractory VT | 34 | M | N/A | Intramyocardial benign fibroma | ADT, CA, SBRT | 25 Gy | 62.2 |  |
| 18 | Hašková et al. Case 3 | Mar 2022 | Refractory VT | 77 | M | N/A | ICM | ADT, CA, SBRT | 25 Gy | 43.4 |  |
| 19 | Li et al. (17) | Apr 2022 | Refractory VT | 54 | M | 32% | NICM | CA | 25 Gy | 74.7 | Real-time image guided treatment |
| 20 | Pavone et al. (18) | Jul 2022 | Refractory VT | 73 | M | 50% | NICM | ADT, CA | 25 Gy | N/A | History of chemical pleurodesis precluding epicardial CA |
| 21 | Nasu et al. (19) | Aug 2022 | Refractory VT | 58 | M | 26% | NICM | CA | 25 Gy | 29.1 | - |
| 22 | Levis et al. (20) | Aug 2022 | Refractory VT | 73 | M | 40% | NICM, prosthetic mitral valve | ADT, CA | 25 Gy | 89 | Cardiac contractility modulation device |
| 23 | Scanavacca et al. (21) | Oct 2022 | Refractory VT | 53 | M | 20% | Chronic Chagas cardiomyopathy | ADT, CA | 25 Gy | 74.5 | Chagas heart disease |
| 24 | Cozzi et al. (22) | Oct 2022 | Refractory VT | 81 | M | 20% | NICM | ADT, CA | 25 Gy | 122.5 | - |
| 25 | Huang et al. (23) | Nov 2022 | Refractory VT | 63 | M | 29% | ICM, aortic valve stenosis | ADT, CA | 12 Gy | 65.8 | Success with lower dose (12 Gy) |
| 26 | Wutzler et al. (24) | Jan 2023 | Refractory VT | 56 | M | 15% | ICM | ADT, CA | 25 Gy | N/A | Patient with VT storm and VF |
| 27 | Mehrhof et al. (25) Case 1 | Feb 2023 | Refractory VT | 54 | M | LVAD | NICM | ADT, CA | 25 Gy | 75.2 | LV assist device (LVAD) |
| 28 | Mehrhof et al.  Case 2 | Feb 2023 | Refractory VT | 61 | M | LVAD | ICM | ADT, CA, bilateral stellate ganglion block | 25 Gy | 134.6 | LV assist device (LVAD) |
| 29 | Keyt et al. (26) | Jun 2023 | Recurrent VT | 75 | M | 46% | NICM, prosthetic mitral and aortic valves | ADT | 25 Gy | 85 | CA not feasible because of mechanical valves and previous sternotomies |
| 30 | Jiwani et al. (27) | Nov 2023 | Refractory VT | 83 | M | 35% | ICM | ADT, CA | 25 Gy | 146.7 | WiSE-CRT (leadless LV endocardial pacing) |
| 31 | Mages et al. (28) | Nov 2023 | Refractory VT | 74 | M | 35% | NICM | ADT, CA | 24 Gy | 49 | Hologram lens for target evaluation and planning |
| 32 | Gupta et al. (29) | Dec 2023 | PVC triggered polymorphic VT | 48 | M | 10-14% | ICM | ADT | 25 Gy | 193 | CA not done due of LV thrombus |
| **Sum-mary** |  | **Jul 2014 to Dec 2023** | **29 sustained VT**  **2 post-infarct VF/polymorphic VT**  **1 metastasis related non-sustained VT** | **62.2±14.1**  **(range 29-83)** | **3 F (9.4%)**  **29 M**  **(90.6%)** | **32.7±14.3**  **(range 12-75)** | **12 ICM**  **11 NICM**  **1 Mixed**  **4 Tumors**  **1 Arrhythmo-genic CM**  **1 Hypertrophic CM**  **1 Chagas**  **1 None** | **30 ADT**  **24 CA**  **3 prior SBRT** | **25 Gy for most studies** | **79.6±61.4**  **(range 3.5-303)** |  |

ADT, antiarrhythmic drug therapy; CA, catheter ablation; CM, cardiomyopathy; ICM, ischemic cardiomyopathy; LV, left ventricular; NICM, non-ischemic cardiomyopathy; SBRT, stereotactic body radiotherapy; VT, ventricular tachycardia; VF, ventricular fibrillation

**Supplementary Table 1B**. Summary of case reports on cardiac SBRT for ventricular tachycardia: cardiac SBRT treatment and adverse outcomes.

| **#** | **First Author** | **VT Episodes (pre- / post-treatment)** | **ATP Episodes (pre- / post-treatment)** | **ICD Shocks**  **(pre- / post-treatment)** | **Follow-up / Survival** | **Time to VT Recurrence** | **LVEF** | **Adverse Events / Safety** |
| --- | --- | --- | --- | --- | --- | --- | --- | --- |
| 1 | Cvek et al. | N/A | N/A | N/A / 0 | 10 days | N/A | 25% | Increase in troponin serum |
| 2 | Loo et al. | 562 in 2 mo / 52 episodes/mo | N/A | 11 per mo / N/A | Died at 9 mo | 8 mo | 24% | No adverse events |
| 3 | Scholz et al. | 67 per wk / 12 per wk | N/A | N/A | N/A | No recurrence | Stable LVEF | No adverse events |
| 4 | Zeng et al. | 189 / 0 | N/A | N/A | N/A | No recurrence | N/A | No adverse events |
| 5 | Bhaskaran et al. | 600 per hour / 0 | N/A | N/A | N/A | N/A | N/A | Segmental pulmonary embolism 3 days post SBRT |
| 6 | Hašková et al. | Incessant / gradual decrease | N/A | N/A | N/A | N/A | Stable LVEF | N/A |
| 7 | Krug et al. | 5 per wk / 1.5 per wk | 110 / 56 | 81.2% decrease | Died at 57 d | N/A | 15% | Increase in cardiac enzymes, nausea |
| 8 | Jumeau et al. | N/A | N/A | N/A | N/A | 18 mo | Stable LVEF | No adverse events |
| 9 | Marti-Almor et al. | Incessant / 0 | N/A | N/A | N/A | 4 mo | Stable LVEF | No adverse events |
| 10 | Park et al. | 10 per d / 0 | N/A | N/A | 6 mo | N/A | 63% | Mild pulmonary fibrosis |
| 11 | Narducci et al. | N/A | N/A | N/A | 3 mo | 2 mo | 30% | N/A |
| 12 | Fiorentino et al. | 24000 per d / 123 | 25 / 1 | 6 / 1 | N/A | 2 mo | 40% | Small area of myocardial necrosis on PET-CT |
| 13 | Gerard et al.  Case 1 | 16 / 1 | N/A | 6 / 0 | 17 mo | 7 mo | N/A | Mild Esophagitis |
| 14 | Gerard et al.  Case 2 | 17 / 0 | N/A | 6 / 0 | 12 mo | No recurrence | N/A | Self-limited pre-syncopal symptoms |
| 15 | Thosani et al. | Incessant / 0 | N/A | N/A | 4-week | No recurrence | N/A | N/A |
| 16 | Hašková et al.  Case 1 | N/A / 0 | N/A | N/A | 32 mo | Continued having VT till 3 mo after SBRT | N/A | No adverse effects |
| 17 | Hašková et al. Case 2 | N/A / 0 | N/A | N/A | 22 mo | No recurrence | N/A | No adverse effects |
| 18 | Hašková et al. Case 3 | N/A / 0 | N/A | N/A | Died at 1 mo | No recurrence | N/A | Progressive heart failure |
| 19 | Li et al. | 37 per d / 2 per d | 30 / 0 | 3 / 0 | 6 mo | Continued having VT episodes | 41% | No adverse effects |
| 20 | Pavone et al. | 4 in 2 mo / 0 | 0 / 0 | 4 / 0 | 6 mo | No recurrence | 45% | No adverse effects |
| 21 | Nasu et al. | N/A / 0 after blanking | N/A / 0 after blanking | N/A / 0 after blanking | Died at 4 mo | No recurrence after blanking | N/A | Progressive heart failure |
| 22 | Levis et al | 7 in 2 mo / 0 | N/A / 0 | N/A / 0 | 23 mo | No recurrence | 28% | No adverse effects |
| 23 | Scanavacca et al. | N/A / 8 | N/A / 7 | N/A / 1 | N/A | N/A | 30% | N/A |
| 24 | Cozzi et al. | 10 ep per d / 0 | N/A | N/A | N/A | No recurrence | N/A | No adverse effects |
| 25 | Huang et al. | 21 /16 | 15 / N/A | 6 / N/A | 16 mo | 6 mo | Gradual increase to 38% at 15 mo | No adverse effects |
| 26 | Wutzler et al. | N/A | N/A | N/A / 0 | 12 mo | No recurrence | 31% | No adverse effects |
| 27 | Mehrhof et al.  Case 1 | Permanent slow VT / mild decrease | N/A | N/A | Died at 5.5 mo | Remained in permanent slow VT | N/A | No adverse effects |
| 28 | Mehrhof et al.  Case 2 | Permanent slow VT / mild decrease | N/A | N/A | Died at 50 d | Remained in permanent slow VT | N/A | No adverse effects |
| 29 | Keyt et al. | 14 / 0 | 12 / 0 | 2 / 0 | 18 mo | No recurrence | N/A | No adverse effects |
| 30 | Jiwani et al. | 263 / 0 | 263 / 0 | 0 / 0 | 9 mo | No recurrence | 30% | No adverse events |
| 31 | Mages et al. | N/A / 0 after blanking period | N/A / 0 after blanking period | N/A / 0 after blanking period | 6 mo | 6 mo | Stable LVEF | No adverse events |
| 32 | Gupta et al. | N/A / 0 | N/A / 0 | N/A / 0 | 18 mo | No recurrence | 35-39% | Trace pericardial effusion along the posterolateral base |
| **Sum-mary** |  | **Elimination:**  **17 (53%)**  **Decrease:**  **10 (31%)**  **No change /Increase: 0**  **Not reported: 5 (16%)** | **Elimination:**  **8 (25%)**  **Decrease:**  **2 (6%)**  **No change /Increase: 0**  **Not reported: 22 (69%)** | **Elimination:**  **12 (38%)**  **Decrease:**  **2 (6%)**  **No change /Increase: 0**  **Not reported: 18 (56%)** | **Died during follow-up: 6/23 (26%)** | **Recurrence/no remission during follow-up: 12/26 (46%)** | **Stable LVEF/ Increase: 16 (50%)**  **Mild decrease ≤5%: 2 (6%)**  **Decrease >5%:**  **1 (3%)**  **Not reported:**  **13 (41%)** | **No adverse events 18/28 (64%)** |

d, days; mo, months; N/A, not available; wk, weeks

**Supplementary Table 2A**. Duration (months) of follow-up and mortality reported in included studies

| Author | N | Avg. follow-up | SD | Median follow-up | IQR | Avg. time-to-death | SD | Median time-to-death | IQR | Deaths  n | Deaths  % |
| --- | --- | --- | --- | --- | --- | --- | --- | --- | --- | --- | --- |
| Cuculich | 5 | 9.8 | 5.0 | 12.0 | 0.0 | 0.8 | - | 0.8 | 0.0 | 1 | 20.0 |
| Robinson* | 17 | 10.1 | 3.9 | 13.0 | 6.0 | 5.8 | 3.1 | 7.0 | 2.3 | 5 | 29.4 |
| Neuwirth | 10 | 21.2 | 3.2 | 22.0 | 5.5 | 22.0 | 3.5 | 24.0 | 3.0 | 3 | 30.0 |
| Lloyd/  Wight | 14 | 5.9 | 6.4 | 4.8 | 6.9 | 0.9 | 1.1 | 0.9 | 0.8 | 2 | 14.3 |
| Gianni* | 5 | 12.0 | 1.4 | 12.0 | 0.0 | 11.0 | 1.4 | 11.0 | 1.0 | 2 | 40.0 |
| Ho L* | 6 | 12.6 | 6.5 | 13.4 | 6.0 | 1.5 | - | 1.5 | 0.0 | 1 | 16.7 |
| Yugo | 3 | 9.3 | 7.2 | 13.0 | 6.5 | 9.3 | 7.2 | 13.0 | 6.5 | 3 | 100.0 |
| Chin | 7 | 10.3 | 7.2 | 9.0 | 5.5 | 11.5 | 3.5 | 11.5 | 2.5 | 2 | 28.6 |
| Ho G | 6 | 7.2 | 4.6 | 7.5 | 7.5 | 2.0 | 1.4 | 2.0 | 1.0 | 2 | 33.3 |
| Carbucicchio | 7 | 6.7 | 4.9 | 8.0 | 8.6 | 4.7 | 5.6 | 2.8 | 5.4 | 3 | 42.9 |
| Lee | 7 | 12.7 | 9.4 | 18.0 | 13.8 | 3.3 | 4.9 | 0.5 | 4.2 | 3 | 42.9 |
| Qian* | 6 | 8.5 | 3.0 | 8.0 | 2.9 | 6.8 | 2.2 | 7.2 | 2.2 | 3 | 50.0 |
| Molon | 6 | 8.7 | 6.6 | 9.0 | 11.2 | 1.0 | - | 1.0 | 0.0 | 1 | 16.7 |
| Aras^†^ | 8 | 8.2 | 4.2 | 9.0 | 6.8 | 4.5 | 1.7 | 4.5 | 3.0 | 4 | 50.0 |
| Ninni* | 17 | 10.9 | 4.5 | 12.5 | 0.0 | 5.0 | 5.9 | 3.2 | 7.2 | 4 | 23.5 |
| Ree | 6 | 11.0 | 2.0 | 12.0 | 0.8 | 9.0 | 2.8 | 9.0 | 2.0 | 2 | 33.3 |
| Amino | 3 | 12.0 | 10.4 | 6.0 | 9.0 | - | - | - | - | 0 | 0.0 |
| Krug | 5 | 6.6 | 5.3 | 6.0 | 10.5 | 1.5 | - | 1.5 | 0.0 | 1 | 20.0 |
| Siklody | 20 | 18.5 | 7.9 | 24.0 | 10.5 | 10.7 | 8.1 | 12.0 | 9.5 | 7 | 35.0 |
| Miszczyk | 11 | 15.4 | 8.0 | 18.0 | 13.1 | 11.5 | 10.5 | 11.0 | 10.5 | 3 | 27.3 |
| Arkles | 14 | 9.3 | 4.6 | 12.0 | 3.8 | 2.7 | 3.1 | 1.7 | 3.6 | 4 | 28.6 |
| Haskova | 17 | 12.1 | 8.7 | 11.5 | 16.5 | 6.1 | 5.6 | 4.6 | 4.4 | 8 | 47.1 |

*Follow-up duration imputed from median/mean follow-up duration

^†^ Exact time information unavailable, upper limit of range of durations was used

**Supplementary Table 2B**. Number of patients with ventricular tachycardia recurrences and time-to-recurrence (months) reported in included studies

| Author | N | VT rec.  n | % rec. | Avg. time-to-rec. | SD | Median time-to-rec. | IQR |
| --- | --- | --- | --- | --- | --- | --- | --- |
| Cuculich | 5 | 3 | 60.0 | 7.5 | 5.4 | 9.0 | 5.2 |
| Neuwirth | 10 | 8 | 80.0 | 8.0 | 6.8 | 5.5 | 6.2 |
| Lloyd/  Wight^†^ | 14 | 9 | 64.3 | 3.0 | 2.3 | 2.1 | 2.0 |
| Gianni | 5 | 5 | 100.0 | 3.6 | 2.0 | 4.5 | 3.0 |
| Ho L | 6 | 3 | 50.0 | 8.0 | 4.4 | 8.2 | 4.3 |
| Yugo | 3 | 3 | 100 | 5.8 | 7.1 | 2.0 | 6.25 |
| Chin | 7 | 6 | 85.7 | 3.1 | 2.5 | 2.2 | 1.5 |
| Molon | 6 | 2 | 33.3 | 3.5 | 2.1 | 3.5 | 1.5 |
| Aras^‡^ | 8 | 7 | 87.5 | 3.6 | 4.0 | 1.5 | 2.2 |
| Ninni^†^ | 17 | 5 | 29.4 | 5.4 | 4.0 | 5.4 | 5.0 |
| Ree^†^ | 6 | 5 | 83.3 | 4.8 | 3.6 | 3.5 | 2.8 |
| Amino^†^ | 3 | 2 | 66.7 | 2.8 | 1.7 | 2.8 | 1.2 |
| Krug | 5 | 2 | 40.0 | 3.0 | - | 3.0 | 0.0 |
| Miszczyk^‡^ | 11 | 8 | 72.7 | 6.8 | 5.0 | 6.0 | 3.8 |
| Haskova^†^ | 17 | 15 | 88.2 | 2.8 | 2.6 | 1.5 | 0.2 |

*For patients with recurrences within blanking period (≤1.5 months), time to recurrence was assumed at 1.5 months (immediately at the end of blanking period)

^†^VT recurrence from first treated episode/ATP therapy

^‡^Exact time information unavailable, upper limit of range of durations was used

**Supplementary Table** **2C**. Number of patients with ICD shocks and time-to-ICD shock (months) reported in included studies

| Author | N | ICD shocks  n | ICD shocks  % | Mean time-to-ICD shock | SD | Median time-to-ICD shock | IQR |
| --- | --- | --- | --- | --- | --- | --- | --- |
| Cuculich | 4 | 1 | 25.0 | 12.0 | - | 12.0 | 0.0 |
| Neuwirth | 10 | 6 | 60.0 | 15.8 | 9.6 | 13.5 | 9.5 |
| Ho L | 6 | 1 | 16.7 | 8.2 | - | 8.2 | 0.0 |
| Yugo | 3 | 1 | 33.3 | 1.5 | - | 1.5 | 0.0 |
| Chin | 7 | 4 | 57.1 | 3.9 | 2.8 | 3.0 | 1.6 |
| Molon | 5 | 2 | 40.0 | 3.5 | 2.1 | 3.5 | 1.5 |
| Aras^†^ | 8 | 6 | 75.0 | 7.2 | 4.1 | 6.0 | 4.5 |
| Ninni | 15 | 2 | 13.3 | 9.1 | 3.3 | 9.1 | 2.4 |
| Amino | 3 | 1 | 33.3 | 4.0 | - | 4.0 | 0.0 |
| Krug | 5 | 1 | 20.0 | 1.5 | - | 1.5 | 0.0 |
| Haskova | 17 | 12 | 70.6 | 2.0 | 1.4 | 1.5 | 0.1 |

*For patients with ICD shocks within blanking period (≤1.5 months), time to shock was assumed at 1.5 months (immediately at the end of blanking period)

^†^Exact time information unavailable, upper limit of range of durations was used

**REFERENCES**

**(For case reports in Supplementary Table 1A-B)**

1. Cvek J, Neuwirth R, Knybel L et al. Cardiac radiosurgery for malignant ventricular tachycardia. Cureus 2014;6.

2. Loo BW, Soltys SG, Wang L et al. Stereotactic Ablative Radiotherapy for the Treatment of Refractory Cardiac Ventricular Arrhythmia. Circulation: Arrhythmia and Electrophysiology 2015;8:748-750.

3. Scholz EP, Seidensaal K, Naumann P, Andre F, Katus HA, Debus J. Risen from the dead: Cardiac stereotactic ablative radiotherapy as last rescue in a patient with refractory ventricular fibrillation storm. HeartRhythm Case Rep 2019;5:329-332.

4. Zeng LJ, Huang LH, Tan H et al. Stereotactic body radiation therapy for refractory ventricular tachycardia secondary to cardiac lipoma: A case report. Pacing Clin Electrophysiol 2019;42:1276-1279.

5. Bhaskaran A, Downar E, Chauhan VS et al. Electroanatomical mapping-guided stereotactic radiotherapy for right ventricular tachycardia storm. HeartRhythm Case Rep 2019;5:590-592.

6. Haskova J, Peichl P, Pirk J, Cvek J, Neuwirth R, Kautzner J. Stereotactic radiosurgery as a treatment for recurrent ventricular tachycardia associated with cardiac fibroma. HeartRhythm Case Reports 2019;5:44-47.

7. Krug D, Blanck O, Demming T et al. Stereotactic body radiotherapy for ventricular tachycardia (cardiac radiosurgery) : First-in-patient treatment in Germany. Strahlenther Onkol 2020;196:23-30.

8. Jumeau R, Vincenti MG, Pruvot E et al. Curative management of a cardiac metastasis from lung cancer revealed by an electrical storm. Clin Transl Radiat Oncol 2020;21:62-65.

9. Marti-Almor J, Jimenez-Lopez J, Rodriguez de Dios N, Tizon H, Valles E, Algara M. Noninvasive ablation of ventricular tachycardia with stereotactic radiotherapy in a patient with arrhythmogenic right ventricular cardiomyopathy. Rev Esp Cardiol (Engl Ed) 2020;73:97-99.

10. Park J-S, Choi Y. Stereotactic Cardiac Radiation to Control Ventricular Tachycardia and Fibrillation Storm in a Patient with Apical Hypertrophic Cardiomyopathy at Burnout Stage: Case Report. J Korean Med Sci 2020;35.

11. Narducci ML, Cellini F, Placidi L et al. Case Report: A Case Report of Stereotactic Ventricular Arrhythmia Radioablation (STAR) on Large Cardiac Target Volume by Highly Personalized Inter- and Intra-fractional Image Guidance. Front Cardiovasc Med 2020;7:565471.

12. Fiorentino A, Di Monaco A, Surgo A et al. Linac-based STereotactic Arrhythmia Radioablation (STAR) of ventricular tachycardia: Case report and literature review. Clin Case Rep 2021;9:362-366.

13. Gerard IJ, Bernier M, Hijal T et al. Stereotactic Arrhythmia Radioablation for Ventricular Tachycardia: Single Center First Experiences. Adv Radiat Oncol 2021;6:100702.

14. Thosani A, Trombetta M, Shaw G, Oh S, Sohn J, Liu E. Stereotactic arrhythmia radioablation for intramural basal septal ventricular tachycardia originating near the His bundle. HeartRhythm Case Rep 2021;7:246-250.

15. Haskova J, Peichl P, Sramko M et al. Case Report: Repeated Stereotactic Radiotherapy of Recurrent Ventricular Tachycardia: Reasons, Feasibility, and Safety. Front Cardiovasc Med 2022;9:845382.

16. Peichl P, Sramko M, Cvek J, Kautzner J. A case report of successful elimination of recurrent ventricular tachycardia by repeated stereotactic radiotherapy: the importance of accurate target volume delineation. Eur Heart J Case Rep 2021;5:ytaa516.

17. Li J, Chen Q, Li G et al. Stereotactic arrhythmia radiotherapy: a case study of real-time image-guided noninvasive treatment for ventricular tachycardia. Quant Imaging Med Surg 2022;12:2607-2615.

18. Pavone C, Scacciavillani R, Narducci ML et al. Successful ventricular tachycardia radioablation in a patient with previous chemical pleurodesis: A case report. Front Cardiovasc Med 2022;9:937090.

19. Nasu T, Toba M, Nekomiya N et al. Successful Application of Stereotactic Body Radiation Therapy for Ventricular Tachycardia Substrate in a Patient With Nonischemic Cardiomyopathy. Am J Cardiol 2022;184:149-153.

20. Levis M, Dusi V, Magnano M et al. A case report of long-term successful stereotactic arrhythmia radioablation in a cardiac contractility modulation device carrier with giant left atrium, including a detailed dosimetric analysis. Front Cardiovasc Med 2022;9:934686.

21. Scanavacca MI, Pisani CF, Salvajoli B et al. Stereotactic Body Radiation Therapy for Recurrent Ventricular Tachycardia in Chagas Disease: First Case in Latin America. Arq Bras Cardiol 2023;120:e20220614.

22. Cozzi S, Bottoni N, Botti A et al. The Use of Cardiac Stereotactic Radiation Therapy (SBRT) to Manage Ventricular Tachycardia: A Case Report, Review of the Literature and Technical Notes. J Pers Med 2022;12.

23. Huang SH, Wu YW, Shueng PW et al. Case report: Stereotactic body radiation therapy with 12 Gy for silencing refractory ventricular tachycardia. Front Cardiovasc Med 2022;9:973105.

24. Wutzler A, Tiedke B, Osman M, Mahrous N, Wurm R. Elimination of refractory ventricular tachycardia storm and fibrillation using stereotactic radiotherapy. Clin Case Rep 2023;11:e6690.

25. Mehrhof F, Bergengruen P, Gerds-Li JH et al. Cardiac radioablation of incessant ventricular tachycardia in patients with terminal heart failure under permanent left ventricular assist device therapy-description of two cases. Strahlenther Onkol 2023;199:511-519.

26. Keyt LK, Atwood T, Bruggeman A et al. Successful Noninvasive 12-Lead ECG Mapping-Guided Radiotherapy of Inaccessible Ventricular Tachycardia Substrate Due to Mechanical Valves. JACC Case Rep 2023;15:101870.

27. Jiwani S, Akhavan D, Reddy M, Noheria A. Cardiac stereotactic radiotherapy for refractory ventricular tachycardia in a patient with wireless left ventricular endocardial stimulation system. HeartRhythm Case Rep 2023;9:818-822.

28. Mages C, Steinfurt J, Rahm AK et al. Recurrent ventricular tachycardia originating from the "left ventricular summit" effectively eliminated by stereotactic irradiation - A case report. HeartRhythm Case Rep 2023;9:802-807.

29. Gupta M, Liu E, Shaw G et al. Stereotactic ablative radiotherapy in ventricular fibrillation with left ventricular thrombus. HeartRhythm Case Rep 2023;9:902-905.
